# Lysine p-nitroanilide impairs cellular energetics and potentiates statin-induced cytotoxicity in RD rhabdomyosarcoma cells

**DOI:** 10.1371/journal.pone.0337895

**Published:** 2025-12-04

**Authors:** Johan Alvarado-Calderón, Guillermo Juárez-Vega, Jesús Rosendo Martínez-Arellano, Hilda Sánchez-Vidal, Imelda Cecilia Zarzoza-Mendoza, David Morales-Morales, Juan Manuel Germán-Acacio, Rogelio Rodríguez-Sotres, Lilia G. Noriega, José Carlos Páez-Franco

**Affiliations:** 1 Red de Apoyo a la Investigación, Universidad Nacional Autónoma de México-CIC, Instituto Nacional de Ciencias Médicas y Nutrición Salvador Zubirán, Ciudad de México, México; 2 Dirección de Investigación, Instituto Nacional de Ciencias Médicas y Nutrición Salvador Zubirán, Ciudad de México, México; 3 Instituto de Química, Universidad Nacional Autónoma de México, Ciudad de México, México; 4 Departamento de Bioquímica, Facultad de Química, Universidad Nacional Autónoma de México, Ciudad de México, México; 5 Departamento de Fisiología de la Nutrición, Instituto Nacional de Ciencias Médicas y Nutrición Salvador Zubirán, Ciudad de México, México; Fujita Health University: Fujita Ika Daigaku, JAPAN

## Abstract

Statins are clinically effective drugs for treating dyslipidemia and have been proposed as promising antineoplastic and adjuvant agents in cancer therapy for years due to their impact on dysregulated cell growth processes, including cell signaling, energetics, and membrane synthesis. Despite being potent inhibitors of mevalonate synthesis and its downstream products, their limited clinical success highlights the need to further explore their mechanistic effects. Leveraging the observed sensitivity of muscle cells to atorvastatin in clinical settings and utilizing untargeted metabolomic analysis of atorvastatin-treated RD rhabdomyosarcoma cells, we identified reduced levels of aminoadipic acid, an intermediate in lysine catabolism. We investigated whether metabolic sensitization of RD cells to lysine-related metabolites (lysine, aminoadipic acid, pipecolic acid, glutamic acid, α-ketoglutarate, and lysine-p-nitroanilide) prior to atorvastatin treatment enhances its cytotoxic effects. Metabolic sensitization or reprogramming involves cellular processes wherein cells adapt their metabolism to environmental changes, reflecting alterations in enzymatic activity, transport, and stress response thresholds. These adaptations enable cells to cope with specific environmental pressures but may impair their ability to respond to other stressors or stimuli. To evaluate the impact of metabolic supplementation, we analyzed cellular stress response markers via western blot. The results revealed that lysine-p-nitroanilide increased BiP, the master regulator of the unfolded protein response, and augmented the phosphorylation at threonine 172 of AMPK, an indicator of altered cellular energetics. Further analysis demonstrated that combining lysine-p-nitroanilide with atorvastatin disrupted mitochondrial homeostasis and reduced glycolysis, both desirable outcomes in antineoplastic treatments. Lysine-p-nitroanilide acts as an in vitro inhibitor of α-aminoadipic semialdehyde synthase, enzyme essential for lysine metabolism via the saccharopine pathway. However, we demonstrated that it is catabolically cleaved to p-nitroanilide, with this molecule driving the cytotoxic activity observed in our experiments. Although lysine metabolism was not fully suppressed by lysine-p-nitroanilide, these findings provide valuable insights for developing novel therapies for rhabdomyosarcoma.

## Introduction

Rhabdomyosarcoma (RMS) is the most common soft tissue sarcoma in children and young adults. It originates from skeletal muscle or other tissues able to differentiate into muscle-like tissue with variable degree of differentiation [1]. Although rare in adults, RMS in this population is associated to a poor prognosis [2]. Multidrug therapy, including chemotherapy, surgery, and radiation, achieves a response in approximately 60% of patients, but metastatic and treatment-resistant cases pose significant challenges to effective cancer therapy [3,4].

*In vitro* models have provided critical insights into anticancer research, enhancing the efficiency of animal and clinical trials [5]. These models serve as a foundation for evaluating novel drugs, combination therapies, and alternative treatments for RMS [6–11]. Vulnerabilities in cancer cells, exposed by recent studies on metabolic reprogramming, can be exploited to enhance cellular sensitivity to treatments [12]. Cellular metabolism adapts to environmental conditions to meet internal energy and nutrient demands during processes such as growth or quiescence. Thus, depending on their genetic background, cells may mobilize structures, organelles, proteins, and/or metabolites in response to prevailing conditions, which also modify their metabolic vulnerability. In essence, preconditioning cancer cells through metabolic stimuli, prior to antitumor drug administration, may enhance treatment efficacy [13]. To this aim, repurposing approved available drugs with an established clinical history offers a cost-effective short-cut to drug development [14].

Statins, widely used in clinical settings, are associated with muscle-related side effects, including weakness, pain, and, in severe cases, rhabdomyolysis, suggesting their potential as therapeutic agents for RMS and other cancers [15,16]. However, despite their effects on membrane synthesis, cell signaling, and energy metabolism, clinical trials with statins have yielded disappointing results [17–19], highlighting the loose connection between promising *in vitro* findings and actual clinical outcomes.

Here, metabolic reprogramming was investigated from the analysis of the metabolic profile of statin-treated cells to spot potential vulnerabilities, which could be exploited to enhance the *in vitro* sensitization of cancer cells to statin treatment. Using a cellular model of atorvastatin-sensitive RD cells combined with untargeted metabolomics (gas chromatography/ mass spectrometry, GC/MS), we observed reduced levels of aminoadipic acid (AA), a lysine catabolism metabolite, in cells exposed to non-cytotoxic statin concentrations. AA is produced from lysine via the pipecolic and/or the saccharopine pathways. The pipecolic pathway takes place in the cytosol and mitochondria, it involves multiple enzymes to generate pipecolate, which is then converted to AA by the 2-aminoadipic acid semialdehyde dehydrogenase (AASD) in mitochondria [20]. In the saccharopine pathway, lysine is processed by the mitochondrial multifunctional enzyme α-aminoadipic semialdehyde synthase (AASS), which has two catalytic sites. The first site condenses lysine with α-ketoglutarate to form saccharopine, while the second site converts saccharopine into glutamate and 2-aminoadipic acid semialdehyde. This last product is then transformed into AA by AASD. Both pathways converge at AASD to produce AA, which can be further metabolized to synthesize Coenzyme A (CoA) [20].

Although lysine metabolism is well-studied in muscle-related processes, such as autophagy and cellular maintenance, the downstream mechanisms of statin-induced sensitization remain unclear. In this work, metabolic reprogramming was tested through pre-incubation with lysine, AA, pipecolic acid, glutamic acid, α-ketoglutarate, or the AASS inhibitor lysine-p-nitroanilide (LPN). The intent was to find if one of these treatments combined with atorvastatin could impair cell viability of an RD model cell line. To elucidate potential mechanisms underlying cytotoxicity, markers of cellular stress, differentiation, autophagy, and apoptosis were evaluated. Notably, LPN was metabolized into p-nitroaniline (PNA) in cells, leading to reduced cell viability compared to other treatments. Although LPN did not effectively inhibit AASS, it increased levels of activated AMP-activated protein kinase (AMPK) and the chaperone BiP, a key regulator of the unfolded protein response (UPR). Further analysis revealed decreased oxygen consumption and basal glycolysis rates, consistent with the desirable properties of antineoplastic agents [21–23].

## Methods

### Reactants

Atorvastatin (PHR1422-Sigma), L-lysine (L5501-Sigma), saccharopine (HY-W040307B- MedChemExpress), aminoadipic acid (A7275-Sigma), p-nitroanilide-Lysine dihydrobromide (L7002-Sigma), pipecolic acid (P2519-Sigma), glutamic acid (G1251-Sigma), alpha-ketoglutaric acid (75890–Sigma), Methoxiamine hydrochloride (89803–Sigma), N,O-Bis(trimethylsilyl)trifluoroacetamide (BSTFA, 15222-Sigma), chlorotrimethylsilane (89595-Sigma), tridecanoic acid (T0502-Sigma), sodium acetate −2-13C (279315-Sigma)

Antibodies: Lc3bI-II (Cell Signaling–3868s), P62 (Cell Signaling–88588), FBX32 (Abcam-Ab168372), AMPK (Cell Signaling–2532), pT172AMPK (Cell Signaling–2535), PARP (Cell Signaling–9542), BiP (Cell Signaling – 3177), P53 (Cell Signaling – 9282), pP53Ser20 (Cell Signaling–9287), Myogenin (Santa Cruz–52903), MyoD1 (Cell Signaling–D8G3), HMGCR (Santa Cruz–271595), RAP1 (Santacruz–398755), Cleaved Caspase-3 -Asp175 (Cell signaling–9661), β-actin (Cell Signaling–4970).

### Cell culture

Rd-18 (CCL136) cells were purchased directly from ATCC. The cells were maintained for 10 passes before renewal. For RD culture maintenance, cells were propagated (85000 cells in 100 mm culture plate) in DMEM—high glucose media (D5648–Sigma) supplemented with10% SFB (16000044–Gibco) for seven days. For metabolomics, western blot (WB) and viability assays, undifferentiated cells were counted with Neubauer chamber (36000 per well to a 6 well or 3400 per well to a 96 plaque) and cultivated with differentiation media (1% SFB) for four days. After this time, the media was renewed, and the metabolic challenge was added. After 24 hours the atorvastatin treatment was applied for 24, 48 or 72 additional hours. To elicit the DNA damage response (DDR) the cells were treated with amsacrine (AMSA 10 µM) and to elicit the UPR the cells were treated with dithiothreitol (DTT 0.5 mM).

### Viability assays—Crystal violet

After differentiation, cell media was renewed, treated, afterwards, the media was removed and 100 µL of glycerdialdehyde 1% were added for 15 min. The liquid was removed and 50 µL of violet crystal (5 mg/mL dissolved in 3:1 methanol/water) was added and the cells were incubated for 30 min. Finally, the crystal violet solution was removed, and the plaque was washed thoroughly with tap water. The precipitate was dissolved with 10% acetic acid solution, mixed in an orbital shaker for 15 min and read at 590 nm in a spectrophotometer (Xmark – BIORAD).

### Cholesterol synthesis inhibition

Differentiated treated cells were incubated with 2-^13^C-acetate 0.75 mM for 24 h and then atorvastatin was added to the media, for an additional 24h. After the incubation period the cells were washed twice with PBS and extracted with ice cold methanol. The extract was centrifuged at 15000 rpm, for 15 min, at 4°C, and the supernatant was transferred to a microcentrifuge tube and dried overnight on a Speedvac system (SPD 121P—Thermo Scientific). The dried pellet was resuspended with MBSTFA 1%TMCS, transferred into a glass vial with an insert and incubated at 37 °C, for 30 min. One µL of this extract was injected into a GC/MS (Agilent 5977A/7890B, Santa Clara, CA, USA) system with an HP5-MS (Agilent) column with helium 99.9999% purity (splitless, flow 1 ml/min, electron ionization, with a range of 50–500 m/z.). A targeted analysis for ions 372–379 m/z related to cholesterol synthesized from 2-^13^C-acetate was performed as described in [24].

### Metabolomic untargeted analysis

Differentiated treated cells were washed twice with 1 mL PBS and the liquid was fully removed by pipetting. Then, 1 mL of ice-cold methanol with 2.5 µL of internal standard consisting of tridecanoic acid (0.1 mg/mL) was added, and bath–sonicated for 2 minutes. The extract was transferred to microcentrifuge tubes, vortexed thoroughly and centrifugated for 10 min, at 15000 rpm, at 4°C. The supernatant was recovered and dried overnight with Speedvac (SPD 121P—Thermo Scientific). Thirty µL of 20 mg/mL methoxamine hydrochloride was added under nitrogen flow and incubated for 90 min, at 37°C. Afterwards, the tube was centrifuged, and the supernatant was recovered and transferred to an insert tube with 30 µL of MBSTFA 1% TMCS, vortexed, and incubated for 30 min. One µL of this extract was injected into the GC/MS system as described in the preceding section. The cromatography consisted of 1 min hold at 60 ◦C with an increased ramp of 10◦C/min to 325 ◦C, with a final held time of 10 min. Raw data was transformed with Agilent Mass hunter to mzdata and deconvolution and alignment was performed with Mzmine2.0. Univariate, multivariate and chemoinformatic analysis were performed with Metaboanalyst (4.0) and Graphpad (V.8.0).

### Protein purification and WB

Treated cells were washed twice with 1 mL PBS, and protein content was extracted with 300 µL of extraction buffer consisting of 10% sodium deoxycholate in 10% TEAB supplemented with protease inhibitors and phosphatase cocktail inhibitors. The extract was heated to 80°C for 5 min and disaggregated through insulin syringe passages. The cell extract was centrifuged at 15000 rpm during 15 min and the supernatant was recovered. Protein quantitation was performed with bicinchoninic acid assay kit (BCA-ThermoScientific-23225) and aliquots were generated for WB. Then, SDS/PAGE was performed, and the protein was transferred onto a nitrocellulose matrix. The membrane was blocked with skimmed milk 10% for 1 hour and it was incubated with the primary antibodies at 1/1000 overnight, followed by incubation with secondary antibodies (Jackson Lab, at 1/2000 dilution) for 1h. The blots were documented in Chemidock (Biorad). Western blots for the qualitative analysis of proposed markers were conducted in duplicate, yielding consistent results. Densitometric analyses were performed in triplicate from three independent samples using ImageJ (U.S. National Institutes of Health).

### Mitochondrial stress test

A total of 4200 cells were seeded per well in a 96-well Seahorse plate using DMEM supplemented with 1% SFB for 96h. At this time, the pre-treatments with PNA (0.1−0.01mM) were added in 10 μL of media and incubated for 24 h. Subsequently, atorvastatin was added at IC50 for 24 h more. Mitochondrial function was evaluated by performing a mitochondrial stress test with an extra injection of 2-deoxyglucose in a XFe96 Extracellular Flux Analyzer (Agilent Technologies). Briefly, cells were washed and incubated for 1 h in a non-CO_2_ incubator with XF DMEM Medium (pH 7.4) supplemented with 25 mM glucose, 1 mM pyruvate and 2 mM glutamine. During the experiment, 1 μM oligomycin, 0.5 μM carbonyl cyanide-p-trifluoromethoxy phenyl-hydrazone (FCCP), 1 μM rotenone/antimycin A and 50 mM of 2-deoxyglucose (2-DG) were injected sequentially, and three measurements were performed in basal conditions and after the addition of each compound. Hoechst 33342 (final 2 μM) was co-injected with the 2-DG to dye the nucleus. At the end of the assay, cell nuclei were counted using Gen5 and Cytation 1 (Biotek). The oxygen consumption rates (OCR) and extracellular acidification rates (ECAR) measurements were normalized to the number of cells. Basal mitochondrial respiration, ATP-linked respiration, proton leak, maximal respiration, non-mitochondrial respiration and spare respiratory capacity were then calculated from the OCR values, and basal glycolysis, glycolytic reserve, glycolytic capacity and non-glycolytic acidification were derived from the ECAR values as previously described [25].

## Results

### Model of muscle statin induced damage

**Fig 1:**
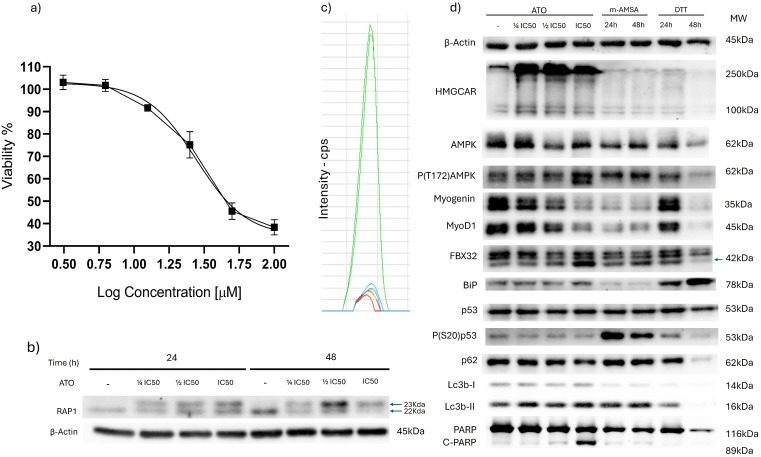
Model of atorvastatin-induced cytotoxicity in RD skeletal muscle cells. **(a)** Determination of the IC50 for RD cells treated with atorvastatin (ATO) for 72h, indicating the concentration required to inhibit 50% of cell viability. **(b)** Electrophoretic mobility assay of RAP1 protein in RD cells treated with atorvastatin at 24 and 48 hours. **(c)** Targeted mass spectrometry analysis of ions in the m/z range of 372–379. The green peak represents cells cultured in media supplemented with 2-¹³C-acetate. The red peak corresponds to cells cultured with 2-¹³C-acetate and treated with atorvastatin (blue peak) for 24h, while the green peak indicates cells grown in standard media without atorvastatin at the same time. **(d)** Western blot (WB) analysis of biomarkers associated with energetic stress (AMPK, pT172-AMPK), cell differentiation (MyoD1, Myogenin), muscle stress (FBX32), protein unfolding or damage (BiP), DNA damage (p53, pSer20-p53), autophagy (p62, Lc3b), and apoptosis (PARP) in RD cells treated with atorvastatin (in IC50 fractions) for 72 h. As positive controls, cells were treated with amsacrine (10 µM) or DTT (0.5 mM) for 24 or 48 hours.

The IC50 for atorvastatin was determined using a crystal violet assay, yielding a value of 27.8 µM ± 1.1 (Fig 1a) after 72h of incubation. To investigate whether atorvastatin altered processes related to mevalonate synthesis in our model, we detected the geranylgeranylation inhibition of Rap1 protein in atorvastatin treated cells through molecular weight mobility shift employing WB (Fig 1b). To confirm that RD cells rely on their own machinery for cholesterol biosynthesis, we performed a 2-^13^C-acetate incorporation assay, followed by targeted analysis of ions in the 372–379 m/z range, which are indicative of cholesterol synthesis from 2-^13^C-acetate. RD cells incubated with this marker produced a peak at 27 min (green peak) confirming de novo cholesterol synthesis (Fig 1c). Statin incubation in media with 2-^13^C-acetate and normal media showed basal signal (red and blue respectively). To evaluate general cellular responses in our model, we conducted WB analysis for apoptosis (PARP cleavage), autophagy (Lc3b, p62), altered energetics (pThr172-AMPK), DNA damage (p-Ser20-p53), UPR (BiP), muscle differentiation (MyoD1, myogenin), and muscle stress (FBX32). Atorvastatin treated cells exhibited a high molecular weight signal on the HMGCR blot; however, this finding may result from HMGCR synthesis intermediates or protein aggregation (the mature HMGCR has a molecular mass of 100 kDa). Atorvastatin at 1 IC50 increased the levels of FBX32, an ubiquitin ligase that promotes the turnover of several proteins under skeletal muscle stress. Additionally, autophagy markers p62 and Lc3b were altered by statin treatment, both are markers of autophagy processes, albeit analysis for increased or decreased autophagy require probing additional protein markers, and the use of certain inhibitors for unequivocal interpretation [26]. In the present work the markers suggest autophagy alteration, but not if this process has increased or decreased. To assess cellular energetics, we analyzed AMPK phosphorylation at threonine 172, which was elevated at higher statin concentrations (1 IC50), indicating a probable altered AMP/ATP ratio. Conversely, atorvastatin reduced levels of myogenic differentiation factors MyoD1 and myogenin and increased PARP cleavage, suggesting a loss of cellular differentiation integrity and possibly, induction of apoptosis. Finally, we confirmed that RD cells can sustain DDR (pSer20-p53) and increase the levels of the UPR modulator BiP when treated with amsacrine (a topoisomerase II inhibitor) and DTT (a disulfide bond reducer), respectively (Fig 1d).

### Metabolomic profiling of atorvastatin treated cells

**Fig 2:**
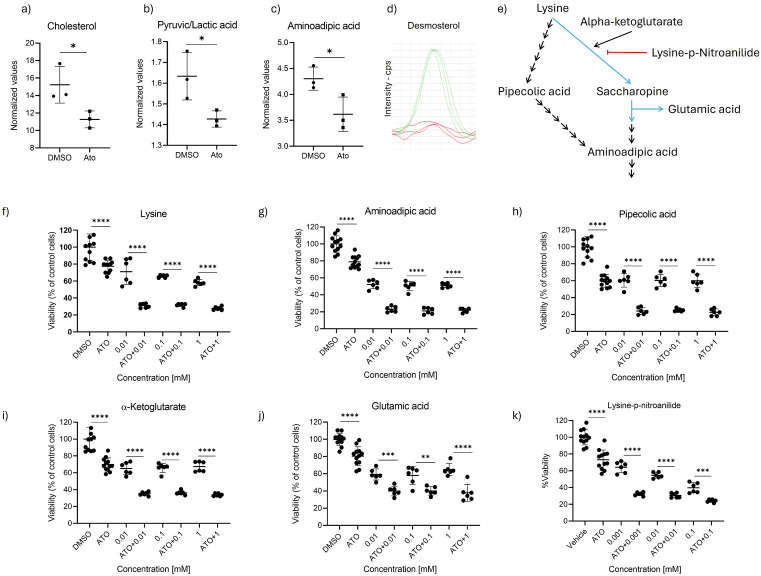
Metabolomic analysis of RD cells treated with non-cytotoxic levels of atorvastatin. Untargeted metabolomic analysis of RD rhabdomyosarcoma cells treated with atorvastatin (ATO) at 1/2 IC50 for 48 hours. **(a-d)** T-test analysis of differentially altered metabolites, with a chromatogram highlighting the desmosterol peak. **(e)** Lysine degradation pathway illustrating metabolite intervention by pre-incubation (24 hours) followed by atorvastatin treatment (½ IC50) for 72 hours. Dashed lines indicate multiple enzymatic steps in the pipecolate pathway; solid blue arrows represent the activity of the bifunctional enzyme AASS. **(f-k)** Viability assays of RD cells pre-incubated with specified metabolites for 24 hours, followed by atorvastatin treatment for 72 hours. Statistical significance was determined using two-tailed t-tests or one-way ANOVA with Dunnett’s post-hoc test. Significance levels: ****p < 0.0001, ***p < 0.001, **p < 0.01, *p < 0.05.

To investigate the minimal metabolic changes induced by atorvastatin prior to cytotoxicity events, we incubated RD cells at half the IC50 concentration (1/2 IC50) for 48 hours. Using our methodological approach, we identified 22 distinct metabolites listed in S1 Table. Unsupervised multivariate principal component analysis (PCA) of these metabolites did not show a clear separation. Thus, cells treated with statin or vehicle seem to share a similar metabolic profile at 48h (S1 Fig). Only cholesterol, desmosterol (not detected in atorvastatin chromatograms and excluded from PCA), AA, and pyruvic/lactic acid peaks exhibited relatively significant changes at this point in time (Fig 2a–2d). Beyond the expected changes, altered pyruvic/lactic acid levels may be linked to the statin’s effect on mitochondrial activity, specifically through inhibition of complex III in the electron transport chain [27] or through ubiquinone synthesis inhibition [28]. On the other hand, the influence of mevalonate pathway inhibition on AA levels, a metabolite produced during lysine catabolism, is less clear. This metabolite is generated via the saccharopine and pipecolate pathways, which converge at the α-aminoadipate-δ-semialdehyde synthase (AASS) bifunctional enzyme, and then it can contribute to the synthesis of other intermediates, such as CoA (Fig 2e). Lower levels of AA could result from reduced lysine transport or altered metabolism. In this context, increased protein synthesis or post-translational modifications may lead to decreased lysine pools. Metabolic rewiring could also divert lysine catabolism to support other pathways [29]. In addition to its primary role, lysine may act as an antioxidant by buffering processes such as mitochondrial function or lactic acid production [20]. To explore the influence of lysine and related compounds over cell viability in this cell model, the cells were pre-incubated for 24h with these metabolites prior to a final incubation with atorvastatin (1/2 IC50) for an additional 72h. Cell viability decreased in all cases (Fig 2f–2k).

### Supplementation with lysine and related metabolites

**Fig 3:**
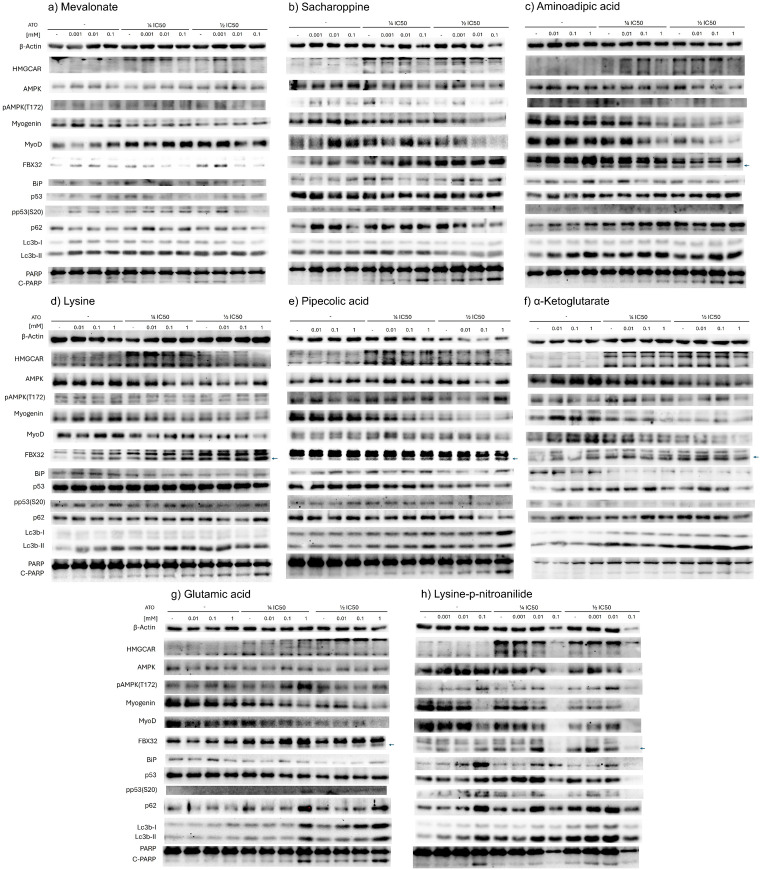
Metabolic supplementation alters western blot stress markers alone or combined with atorvastatin. Representative Western blots for **(a)** mevalonate, **(b)** saccharopine, **(c)** aminoadipic acid, **(d)** lysine, **(e)** pipecolic acid, **(f)** α-ketoglutarate, **(g)** glutamic acid, and **(h)** lysine-p-nitroanilide (LPN) treatments (24 h), followed by atorvastatin treatment for 72 hours. All experiments were performed in duplicate from two independent experiments.

To investigate the cytotoxic effects observed with metabolite pre-incubation, we qualitatively explored the proposed markers shown in Fig 1d. As controls, we used mevalonate, an immediate metabolite of HMGCR activity, and saccharopine, a known, potentially toxic intermediate, of lysine catabolism [30,31]. Pre-incubation with mevalonate restored FBX32 and PARP to non-cytotoxic levels (Fig 3a). Pre-incubation with saccharopine, a mitochondrial metabolite, relevant to mitochondrial homeostasis in various tissues [31–33], increased PARP cleavage even at lowest concentration tested (0.001 mM), but only in cells treated with atorvastatin (¼ & ½ IC50). FBX32 expression was induced both as a monotherapy and in combination with atorvastatin, correlating with reduced levels of differentiation markers MyoD1 and myogenin (Fig 3b). Notably, despite elevated FBX32 levels with saccharopine monotherapy, autophagy markers seem to remain unaffected (Lc3b and p62, Fig 3b). In contrast, pre-incubation with AA altered autophagy markers (Lc3b and p62 Fig 3c) and enhanced PARP cleavage without apparent FBX32 accumulation, while reducing MyoD1 and myogenin levels. Lysine pre-incubation increased FBX32 expression as monotherapy and further elevated this stress marker when combined with atorvastatin (Fig 3d). PARP cleavage was enhanced at ½ IC50 in combination with lysine, accompanied by decreased myogenin levels. Pipecolate supplementation similarly increased PARP cleavage, and when at 1 mM and combined with atorvastatin (½ IC50), it enhanced AMPK phosphorylation and Lc3b signals (Fig 3e). Alpha-ketoglutarate pre-incubation did not alter PARP cleavage but reduced RD cell viability (Fig 2f). Glutamic acid at 1 mM increased AMPK phosphorylation, altered autophagy markers, elevated FBX32 expression, and enhanced PARP cleavage when combined with atorvastatin at ¼ IC50 (Fig 3g).

**Fig 4:**
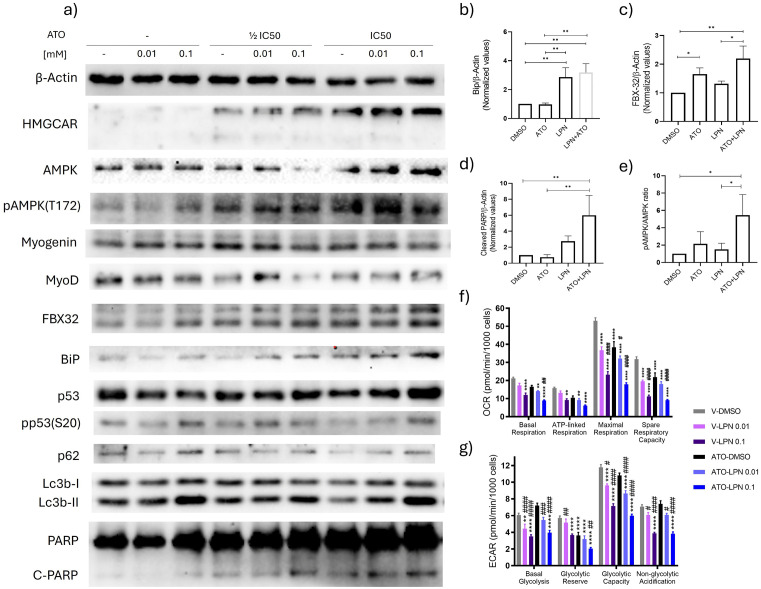
LPN alters stress WB stress markers, OCR and ECAR parameters Western blot analysis of stress markers in RD cells treated with atorvastatin for 24 hours, with or without 0.01–0.1 mM LPN pre-treatment for 24 hours (a). Densitometric analysis of BiP, FBX32, cleaved PARP, and Thr172-pAMPK levels in RD cells with or without 0.1 mM LPN pre-treatment for 24 hours. Densitometric analyses were conducted using ImageJ software, and bar graphs represent the mean ± SD of three independent replicates. Control levels were normalized to 1, and only statistically significant changes are shown **(b-e)**. Mitochondrial respiration parameters in RD cells treated with 0.01–0.1 mM LPN, with or without atorvastatin at its IC50, for 24 hours **(f)**. Glycolysis parameters in RD cells under the same conditions **(g)**. Oligomycin-O, carbonyl cyanide-p-trifluoromethoxy phenyl-hydrazone-FCCP, rotenone/antimycin-R/A and 2-deoxyglucose- 2-DG. Statistical differences were assessed by one-way ANOVA followed by Tukey multiple comparison post hoc test. Significance levels compared to control: ****p < 0.0001, ***p < 0.001, **p < 0.01, *p < 0.05. Significance levels compared to atorvastatin treatment: ####p < 0.0001, ###p < 0.001, ##p < 0.01, #p < 0.05.

LPN, an in vitro L-lysine-α-ketoglutarate reductase inhibitor [34], induced cell death as a monotherapy at a low concentration (0.001 mM), and the effect was increased by atorvastatin treatment (Fig 2k,3h and 4a). Densitometric analysis revealed that LPN when combined with atorvastatin increased BiP expression, suggesting UPR activation [23,35,36] (Fig 4b). However, additional markers such as IRE1-alpha activation or ATF6 nuclear translocation, are needed to confirm this hypothesis [37]. It also resulted in increased FBX32 protein and PARP cleavage when combined with atorvastatin at 24 (Fig 4c,4d). In addition, it enhanced AMPK phosphorylation at threonine 172, indicating potential dysregulation of cellular energetics (Fig 4e).

To test this hypothesis, OCR and ECAR were measured in RD cells treated for 48 hours with LPN (0.01–0.1 mM), alone or in combination with atorvastatin at 1 IC50, for 24h. LPN significantly reduced basal (0.1mM), ATP-linked (0.1mM), and maximal respiration rates (0.01 and 0.1 mM) compared to controls (Fig 4f). Treatment with atorvastatin alone decreased maximal respiration and spare respiratory capacity relative to controls (Fig 4f). However, pre-incubation with LPN in atorvastatin-treated cells did not produce an additive effect on these parameters. In contrast, basal glycolysis was reduced by LPN at both concentrations (0.01 and 0.1 mM) (Fig 4g). Atorvastatin alone decreased glycolytic reserve, which was further reduced when combined with LPN at 0.1 mM. Glycolytic capacity and non-glycolytic acidification were also diminished by LPN monotherapy and remained at these levels in combination with atorvastatin (Fig 4g).

### LPN metabolization

**Fig 5:**
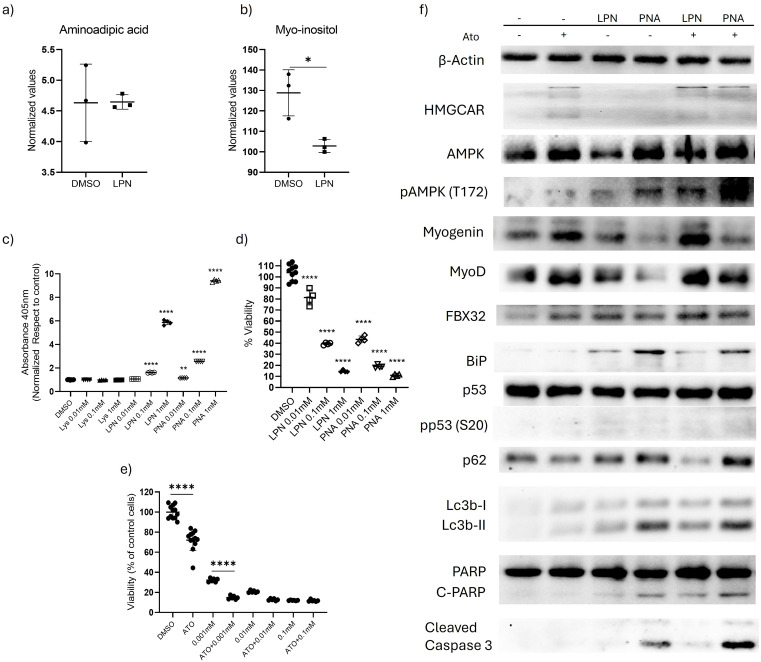
LPN is metabolized to PNA and alters WB stress markers. T-test analysis of **(a)** aminoadipic acid and **(b)** myo-inositol levels in RD cells treated with 0.01 mM LPN for 48 hours. Stability analysis of LPN based on absorbance at 405 nm in RD cells after 72 hours of incubation **(c)**. Viability assays using crystal violet staining in RD cells treated with LPN or PNA for 72 hours **(d)**. Viability assays of RD cells pre-incubated with PNA for 24 hours followed by atorvastatin treatment (1/2 IC50) for 72 hours **(e)**. Western blot analysis of RD cells treated with LPN or PNA [0.01mM] for 24 hours followed by atorvastatin treatment for 48 hours **(f)**. Statistical differences between treatments and controls were assessed using one-way ANOVA followed by Dunnett’s post-hoc test. Significance levels: ****p < 0.0001, ***p < 0.001, **p < 0.01, *p < 0.05.

Although LPN induced significant changes in stress markers and cellular energetics, it did not reduce AA levels; only myo-inositol was decreased employing untargeted metabolomic analysis (Fig 5a, 5b). Several possibilities may explain this, including physical or metabolic degradation of LPN. LPN is known to serve as a substrate for aminopeptidase activity from protein samples *in vitro*, where it transforms it to lysine and PNA. This molecule may contribute to the effects observed in our experiments, as reported by Corti, et al. [38]. To determine whether cells metabolize LPN to PNA, RD cells were incubated with LPN for 72 hours, and absorbance at 405 nm was measured. Increased absorbance was consistent with PNA production (Fig 5c). No residual peptidase activity or reduced LPN stability was detected in cell-free media (S2 Fig).

PNA induced decreased viability in RD cells (Fig 5d), and, like LPN, enhanced the cytotoxicity of atorvastatin-treated RD cells (Fig 5e). WB analysis of PNA treated RD cells (alone or combined with atorvastatin) shows stronger effects in pAMPK, BiP, Lc3b, and PARP cleavage than LPN. Additionally, we included cleaved caspase 3 showing an effect (Fig 5f) consistent with the reduction in cell viability.

## Discussion

Cholesterol is essential for cells to synthesize membrane components and produce various metabolites. Cells employ multiple strategies to acquire cholesterol, either by de novo synthesis or importing it from external sources [39]. Statins, widely used for their pharmacological benefits in treating dyslipidemia, also show potential in cancer therapy by inhibiting processes critical for uncontrolled cell growth, including membrane synthesis, energy metabolism, and cell signaling [15]. Although inhibition of HMG-CoA reductase, a key enzyme in the mevalonate pathway, has been explored as a cancer therapy target for decades, clinical trials have largely failed to yield significant results [15]. But further research is needed to determine whether mevalonate synthesis inhibition can be an effective antineoplastic adjuvant to enhance therapeutic efficacy.

The metabolic profile of cells reflects the transport, diffusion, and stability (enzymatic or physical) of metabolites. Although its interpretation is not clear cut, as multiple metabolites share common pathways, this profile provides valuable insights into cellular homeostasis and adaptive responses. Nevertheless, while supplementing specific metabolic intermediates to intervene targeted pathways requires rigorous validation through experiments such as metabolic flux analysis [40], metabolite supplementation—acting as substrates, products, or allosteric regulators—can provide useful hints when combined with the assessment of observables, such as cell growth, stress, or death [41],

In this study, we established a cellular model of RMS with the capacity for endogenous cholesterol synthesis and implemented a panel of viability and stress markers to monitor cellular responses to modified media prior to atorvastatin exposure.

To explore metabolic pathways affected by atorvastatin beyond the mevalonate pathway and independently of cytotoxic effects, we treated cells with a low-cytotoxic dose of atorvastatin. Under these conditions, we observed reduced levels of aminoadipic acid, suggesting a probable impact of lysine catabolism on cholesterol homeostasis in muscle.

Reduced levels of AA may result from decreased lysine internalization, increased AA externalization, downregulated activity of AASS, reduced pipecolic acid levels (due to enzymatic or chemical equilibrium), or a combination of these processes [20,42,43]. Although atorvastatin primarily inhibits HMG-CoA reductase in the endoplasmic reticulum [44], its effects on ubiquinone synthesis and/or accumulation in mitochondria [27,30] may reduce the activity of mitochondrial enzymes, such as AASS, which often function as complexes [45,46]. Clinical studies of statin-treated patients have reported elevated serum levels of lysine and AA [47,48], while in rats, the cytotoxic combination of atorvastatin and fenofibrates (PPARγ inhibitors) reduces serum lysine levels [49].

To investigate the role of the AA pathway in our RMS model, we supplemented cells with lysine, AA, pipecolic acid, glutamic acid, alpha-ketoglutarate, and LPN (an in vitro AASS inhibitor). These interventions generally reduced cell viability and increased apoptosis. However, analyzing markers such as Lc3b, p62, FBX32, MyoD1, and myogenin is not simple, due to their context-dependent regulation.

Skeletal muscle supports motility, structural integrity, and metabolic storage [50], with its functions finely regulated by the balance of tissue synthesis, maintenance, and degradation [51]. Cachexia and sarcopenia, characterized by muscle loss due to aging, disuse, inflammation, or adaptive responses, are primarily driven by autophagy and the ubiquitin-proteasome system [17,52,53]. In this context, lysine metabolism influences protein turnover systems, but its effects vary depending on cellular conditions.

C2C12 cells exposed to high-glucose stress and supplemented with lysine exhibit increased autophagy markers, correlating with reduced cytotoxicity compared to controls [54]. In contrast, C2C12 myotubes treated with lysine and AA show decreased autophagy, associated with reduced protein degradation [55]. Similarly, lysine supplementation in rats fed with a low-protein diet reduces skeletal muscle autophagy, correlating with decreased muscle mass loss [56]. In our RMS cell model, supplementation with atorvastatin, lysine, AA, or LPN altered autophagy markers (p62 and Lc3b) (Fig 3). However, lysine metabolites did not restore cell viability, suggesting that RD cells under these culture conditions may have a reduced stress response capacity or that cholesterol requirements inherently limit viability. Although cholesterol synthesis inhibition typically promotes autophagy [57,58], it was insufficient to restore viability in our model.

FBX32, an E3 ubiquitin ligase involved in stress-induced protein turnover via proteasomal degradation, is directly linked to statin-induced myotoxicity [59,60]. FBX32 downregulates MyoD1 levels through proteasomal degradation [61], which may explain their inverse correlation in our experiments (Fig 3). Once apoptosis is activated (evidenced by PARP cleavage), both FBX32 and MyoD1 levels decrease across all treatments. Notably, differentiation factors like MyoD1, used here as markers of cell integrity, can also signal pro-apoptotic pathways during in vivo muscle differentiation [62–64].

Among the treatments tested, LPN and saccharopine induced FBX32 accumulation at the lowest concentration tested (0.001 mM) (Fig 3). Unlike other pretreatments, LPN also increased BiP and AMPK phosphorylation, a chaperone associated to UPR [65,66] and a messenger of altered cellular energetics [67], respectively (Fig 3h and 4a).

A link between atrogin-1 and BiP regulation has been demonstrated in skeletal muscle, where disruption of this balance induces mitochondrial damage and apoptosis [35]. In our model, this correlates with LPN supplementation. However, LPN is unlikely to act as an AASS inhibitor in our model due to its instability caused by RD cell aminopeptidase activity. Despite this, LPN pretreatment induced the highest cytotoxicity among all tested combinations.

PNA may contribute to the observed effects, as its incubation yields a cytotoxic and marker profile similar to LPN, though it appears more potent at equivalent doses. LPN requires enzymatic cleavage to produce PNA, with PNA levels governed by the kinetic parameters of RD cell aminopeptidases. In *Salmonella typhimurium*, PNA incubation with flavin mononucleotide increases mutagenicity [68]. In A549 cells, PNA supplementation elevates reactive oxygen species and cytotoxicity, effects reversed by N-acetylcysteine (NAC) as an antioxidant [38]. In humans, mice, and rats, PNA supplementation primarily causes methemoglobin formation due to altered redox balance [69,70], which may explain the main effects observed in our model.

Metabolic rewiring of glucose to NADPH synthesis via the pentose phosphate pathway (PPP) [71] may account for the reduced glycolysis and myoinositol levels observed in LPN-treated cells. These PPP effects could be regulated by AMPK activation [72]. As reported by Liu et al., uncontrolled ROS in thyroid cancer cells can trigger the UPR and apoptosis, potentially managed by PPP activity [73].

The mechanism of action of LPN and PNA remains unclear in our data; however, their effects on glycolysis downregulation and protein damage align with desirable outcomes for antineoplastic agents [21–23]. The cleavage of LPN to PNA and lysine requires metabolically active cells, implying that cytotoxicity can potentially be controlled through amino acid transport systems. In this regard, the heightened requirement of cancer cells for amino acid backbones to sustain unrestricted growth and metabolism can be exploited to enhance selectivity for cancerous cells [74].

LPN could be technologically refined to overcome current limitations. In a physiological context, bloodstream aminopeptidases could degrade LPN, potentially causing off-target effects such as methemoglobinemia in other cells and tissues [69]. Developing novel amino acid derivatives containing the PNA moiety, designed to resist bloodstream aminopeptidase activity but undergo effective intracellular cleavage, could enhance selectivity. Leveraging the specific amino acid requirements of proliferating cells may offer innovative strategies for antineoplastic drug development [75].

## Conclusions

We developed a method to evaluate the impact of metabolic reprogramming on stress, differentiation, and apoptosis markers in RMS cells. LPN and PNA, alone or in combination with atorvastatin, increased RD cell cytotoxicity, an effect associated with reduced glycolysis and mitochondrial disruption.

## Supporting information

S1 Table List of metabolites included in the untargeted metabolomics analysis. (DOCX)

S1 Fig Principal component analysis (PCA) comparing atorvastatin-treated and vehicle-treated RD cells (DMSO). (TIF)

S2 Fig Stability assay of lysine, LPN, or PNA in cell culture medium. Measures were based on absorbance at 405 nm over 72 hours. Statistical significance compared to the control was determined using one-way ANOVA followed by Dunnett’s test: ****p < 0.001.
(TIF)

S1 File Minimal data set. (ZIP)

## References

[1] Skapek SX, Ferrari A, Gupta AA, Lupo PJ, Butler E, Shipley J, et al. Rhabdomyosarcoma. Nat Rev Dis Primers. 2019;5(1):1. doi: 10.1038/s41572-018-0051-2 30617281 PMC7456566

[2] Ruiz-Mesa C, Goldberg JM, Coronado Munoz AJ, Dumont SN, Trent JC. Rhabdomyosarcoma in Adults: New Perspectives on Therapy. Curr Treat Options in Oncol. 2015;16(6). doi: 10.1007/s11864-015-0342-8 25975442

[3] Hettmer S, Li Z, Billin AN, Barr FG, Cornelison DDW, Ehrlich AR, et al. Rhabdomyosarcoma: current challenges and their implications for developing therapies. Cold Spring Harb Perspect Med. 2014;4(11):a025650. doi: 10.1101/cshperspect.a025650 25368019 PMC4208704

[4] Miwa S, Yamamoto N, Hayashi K, Takeuchi A, Igarashi K, Tsuchiya H. Recent Advances and Challenges in the Treatment of Rhabdomyosarcoma. Cancers (Basel). 2020;12(7):1758. doi: 10.3390/cancers12071758 32630642 PMC7409313

[5] Sharma SV, Haber DA, Settleman J. Cell line-based platforms to evaluate the therapeutic efficacy of candidate anticancer agents. Nat Rev Cancer. 2010;10(4):241–53. doi: 10.1038/nrc2820 20300105

[6] Konieczny P, Sułkowski M, Badyra B, Kijowski J, Majka M. Suicide gene therapy of rhabdomyosarcoma. Int J Oncol. 2017;50(2):597–605. doi: 10.3892/ijo.2016.3824 28035376

[7] van Erp AEM, Versleijen-Jonkers YMH, van der Graaf WTA, Fleuren EDG. Targeted Therapy-based Combination Treatment in Rhabdomyosarcoma. Mol Cancer Ther. 2018;17(7):1365–80. doi: 10.1158/1535-7163.MCT-17-1131 29967215

[8] Salerno M, Avnet S, Bonuccelli G, Hosogi S, Granchi D, Baldini N. Impairment of lysosomal activity as a therapeutic modality targeting cancer stem cells of embryonal rhabdomyosarcoma cell line RD. PLoS One. 2014;9(10):e110340. doi: 10.1371/journal.pone.0110340 25329465 PMC4203792

[9] Chen X, Stewart E, Shelat AA, Qu C, Bahrami A, Hatley M, et al. Targeting oxidative stress in embryonal rhabdomyosarcoma. Cancer Cell. 2013;24(6):710–24. doi: 10.1016/j.ccr.2013.11.002 24332040 PMC3904731

[10] Stewart E, McEvoy J, Wang H, Chen X, Honnell V, Ocarz M, et al. Identification of Therapeutic Targets in Rhabdomyosarcoma through Integrated Genomic, Epigenomic, and Proteomic Analyses. Cancer Cell. 2018;34(3):411–426.e19. doi: 10.1016/j.ccell.2018.07.012 30146332 PMC6158019

[11] Xu N, Hua Z, Ba G, Zhang S, Liu Z, Thiele CJ, et al. The anti-tumor growth effect of a novel agent DMAMCL in rhabdomyosarcoma in vitro and in vivo. J Exp Clin Cancer Res. 2019;38(1). doi: 10.1186/s13046-019-1107-1 PMC6408795 30850026

[12] Faubert B, Solmonson A, DeBerardinis RJ. Metabolic reprogramming and cancer progression. Science. 2020;368(6487):eaaw5473. doi: 10.1126/science.aaw5473 32273439 PMC7227780

[13] van den Boogaard WMC, van den Heuvel-Eibrink MM, Hoeijmakers JHJ, Vermeij WP. Nutritional Preconditioning in Cancer Treatment in Relation to DNA Damage and Aging. Annu Rev Cancer Biol. 2021;5:161–79. doi: 10.1146/annurev-cancerbio-060820-090737 35474917 PMC9037985

[14] Beloribi-Djefaflia S, Vasseur S, Guillaumond F. Lipid metabolic reprogramming in cancer cells. Oncogenesis. 2016;5(1):e189. doi: 10.1038/oncsis.2015.49 26807644 PMC4728678

[15] Jiang W, Hu J-W, He X-R, Jin W-L, He X-Y. Statins: a repurposed drug to fight cancer. J Exp Clin Cancer Res. 2021;40(1):241. doi: 10.1186/s13046-021-02041-2 34303383 PMC8306262

[16] Turner RM, Pirmohamed M. Statin-Related Myotoxicity: A Comprehensive Review of Pharmacokinetic, Pharmacogenomic and Muscle Components. J Clin Med. 2019;9(1):22. doi: 10.3390/jcm9010022 31861911 PMC7019839

[17] Longo J, van Leeuwen JE, Elbaz M, Branchard E, Penn LZ. Statins as Anticancer Agents in the Era of Precision Medicine. Clin Cancer Res. 2020;26(22):5791–800. doi: 10.1158/1078-0432.CCR-20-1967 32887721

[18] Krishnamurthy N, Grimshaw AA, Axson SA, Choe SH, Miller JE. Drug repurposing: a systematic review on root causes, barriers and facilitators. BMC Health Serv Res. 2022;22(1):970. doi: 10.1186/s12913-022-08272-z 35906687 PMC9336118

[19] Di Bello E, Zwergel C, Mai A, Valente S. The Innovative Potential of Statins in Cancer: New Targets for New Therapies. Front Chem. 2020;8:516. doi: 10.3389/fchem.2020.00516 32626692 PMC7312214

[20] Matthews DE. Review of Lysine Metabolism with a Focus on Humans. J Nutr. 2020;150(Suppl 1):2548S–2555S. doi: 10.1093/jn/nxaa224 33000162

[21] Chen Z, Lu W, Garcia-Prieto C, Huang P. The Warburg effect and its cancer therapeutic implications. J Bioenerg Biomembr. 2007;39(3):267–74. doi: 10.1007/s10863-007-9086-x 17551814

[22] Ojha R, Amaravadi RK. Targeting the unfolded protein response in cancer. Pharmacol Res. 2017;120:258–66. doi: 10.1016/j.phrs.2017.04.003 28396092 PMC5542584

[23] Bonsignore G, Martinotti S, Ranzato E. Endoplasmic Reticulum Stress and Cancer: Could Unfolded Protein Response Be a Druggable Target for Cancer Therapy? Int J Mol Sci. 2023;24(2):1566. doi: 10.3390/ijms24021566 36675080 PMC9865308

[24] Müller C, Junker J, Bracher F, Giera M. A gas chromatography-mass spectrometry-based whole-cell screening assay for target identification in distal cholesterol biosynthesis. Nat Protoc. 2019;14(8):2546–70. doi: 10.1038/s41596-019-0193-z 31341291

[25] Torres A, Noriega LG, Delgadillo-Puga C, Tovar AR, Navarro-Ocaña A. Caffeoylquinic Acid Derivatives of Purple Sweet Potato as Modulators of Mitochondrial Function in Mouse Primary Hepatocytes. Molecules. 2021;26(2):319. doi: 10.3390/molecules26020319 33435516 PMC7827015

[26] Jiang P, Mizushima N. LC3- and p62-based biochemical methods for the analysis of autophagy progression in mammalian cells. Methods. 2015;75:13–8. doi: 10.1016/j.ymeth.2014.11.021 25484342

[27] Schirris TJJ, Renkema GH, Ritschel T, Voermans NC, Bilos A, van Engelen BGM, et al. Statin-Induced Myopathy Is Associated with Mitochondrial Complex III Inhibition. Cell Metab. 2015;22(3):399–407. doi: 10.1016/j.cmet.2015.08.002 26331605

[28] Vaughan RA, Garcia-Smith R, Bisoffi M, Conn CA, Trujillo KA. Ubiquinol rescues simvastatin-suppression of mitochondrial content, function and metabolism: implications for statin-induced rhabdomyolysis. Eur J Pharmacol. 2013;711(1–3):1–9. doi: 10.1016/j.ejphar.2013.04.009 23624330

[29] Leandro J, Houten SM. The lysine degradation pathway: Subcellular compartmentalization and enzyme deficiencies. Mol Genet Metab. 2020;131(1–2):14–22. doi: 10.1016/j.ymgme.2020.07.010 32768327

[30] Mollazadeh H, Tavana E, Fanni G, Bo S, Banach M, Pirro M, et al. Effects of statins on mitochondrial pathways. J Cachexia Sarcopenia Muscle. 2021;12(2):237–51. doi: 10.1002/jcsm.12654 33511728 PMC8061391

[31] Zhou J, Wang X, Wang M, Chang Y, Zhang F, Ban Z, et al. The lysine catabolite saccharopine impairs development by disrupting mitochondrial homeostasis. Journal of Cell Biology. 2018;218(2):580–97. doi: 10.1083/jcb.201807204 30573525 PMC6363459

[32] Guo Y, Wu J, Wang M, Wang X, Jian Y, Yang C, et al. The Metabolite Saccharopine Impairs Neuronal Development by Inhibiting the Neurotrophic Function of Glucose-6-Phosphate Isomerase. J Neurosci. 2022;42(13):2631–46. doi: 10.1523/JNEUROSCI.1459-21.2022 35135854 PMC8973428

[33] Wen J, Feng Y, Xue L, Yuan S, Chen Q, Luo A, et al. High-fat diet-induced L-saccharopine accumulation inhibits estradiol synthesis and damages oocyte quality by disturbing mitochondrial homeostasis. Gut Microbes. 2024;16(1):2412381. doi: 10.1080/19490976.2024.2412381 39410876 PMC11485700

[34] Fjellstedt TA, Robinson JC. Purification and properties of L-lysine-alpha-ketoglutarate reductase from human placenta. Arch Biochem Biophys. 1975;168(2):536–48. doi: 10.1016/0003-9861(75)90285-4 1169916

[35] Ruparelia AA, Montandon M, Merriner J, Huang C, Wong SFL, Sonntag C, et al. Atrogin-1 promotes muscle homeostasis by regulating levels of endoplasmic reticulum chaperone BiP. JCI Insight. 2024;9(8):e167578. doi: 10.1172/jci.insight.167578 38530354 PMC11141880

[36] Verfaillie T, Salazar M, Velasco G, Agostinis P. Linking ER Stress to Autophagy: Potential Implications for Cancer Therapy. Int J Cell Biol. 2010;2010:930509. doi: 10.1155/2010/930509 20145727 PMC2817393

[37] Samali A, Fitzgerald U, Deegan S, Gupta S. Methods for monitoring endoplasmic reticulum stress and the unfolded protein response. Int J Cell Biol. 2010;2010:830307. doi: 10.1155/2010/830307 20169136 PMC2821749

[38] Corti A, Dominici S, Piaggi S, Belcastro E, Chiu M, Taurino G, et al. γ-Glutamyltransferase enzyme activity of cancer cells modulates L-γ-glutamyl-p-nitroanilide (GPNA) cytotoxicity. Sci Rep. 2019;9(1):891. doi: 10.1038/s41598-018-37385-x 30696905 PMC6351548

[39] Huang B, Song B-L, Xu C. Cholesterol metabolism in cancer: mechanisms and therapeutic opportunities. Nat Metab. 2020;2(2):132–41. doi: 10.1038/s42255-020-0174-0 32694690

[40] Antoniewicz MR. A guide to metabolic flux analysis in metabolic engineering: Methods, tools and applications. Metab Eng. 2021;63:2–12. doi: 10.1016/j.ymben.2020.11.002 33157225

[41] Fell D. Understanding the control of metabolism. 1997.

[42] Wang C, Calcutt MW, Ferguson JF. Knock-Out of DHTKD1 Alters Mitochondrial Respiration and Function, and May Represent a Novel Pathway in Cardiometabolic Disease Risk. Front Endocrinol. 2021;12. doi: 10.3389/fendo.2021.710698 PMC8414881 34484123

[43] Shi W, Yang Z, Fu P, Yang Y. Metabolite 2-aminoadipic acid: implications for metabolic disorders and therapeutic opportunities. Front Pharmacol. 2025;16:1569020. doi: 10.3389/fphar.2025.1569020 40432896 PMC12106402

[44] Shi Q, Chen J, Zou X, Tang X. Intracellular Cholesterol Synthesis and Transport. Front Cell Dev Biol. 2022;10:819281. doi: 10.3389/fcell.2022.819281 35386193 PMC8978673

[45] Clark JB, Land JM. Enzymes. Enzymes. 1979;7:351–73. 10.1016/0163-7258(79)90036-6 392558

[46] Kuznetsov AV, Margreiter R, Ausserlechner MJ, Hagenbuchner J. The Complex Interplay between Mitochondria, ROS and Entire Cellular Metabolism. Antioxidants (Basel). 2022;11(10):1995. doi: 10.3390/antiox11101995 36290718 PMC9598390

[47] Fernandes Silva L, Ravi R, Vangipurapu J, Laakso M. Metabolite Signature of Simvastatin Treatment Involves Multiple Metabolic Pathways. Metabolites. 2022;12(8):753. doi: 10.3390/metabo12080753 36005625 PMC9414498

[48] Trupp M, Zhu H, Wikoff WR, Baillie RA, Zeng Z-B, Karp PD, et al. Metabolomics reveals amino acids contribute to variation in response to simvastatin treatment. PLoS One. 2012;7(7):e38386. doi: 10.1371/journal.pone.0038386 22808006 PMC3392268

[49] Strauss V, Mellert W, Wiemer J, Leibold E, Kamp H, Walk T, et al. Increased toxicity when fibrates and statins are administered in combination--a metabolomics approach with rats. Toxicol Lett. 2012;211(2):187–200. doi: 10.1016/j.toxlet.2012.03.798 22484644

[50] Mukund K, Subramaniam S. Skeletal muscle: A review of molecular structure and function, in health and disease. Wiley Interdiscip Rev Syst Biol Med. 2020;12(1):e1462. doi: 10.1002/wsbm.1462 31407867 PMC6916202

[51] Tan KT, Ang S-TJ, Tsai S-Y. Sarcopenia: Tilting the Balance of Protein Homeostasis. Proteomics. 2020;20(5–6):e1800411. doi: 10.1002/pmic.201800411 31722440

[52] Ragni M, Fornelli C, Nisoli E, Penna F. Amino Acids in Cancer and Cachexia: An Integrated View. Cancers. 2022;14(22):5691. doi: 10.3390/cancers14225691 36428783 PMC9688864

[53] Bowen TS, Schuler G, Adams V. Skeletal muscle wasting in cachexia and sarcopenia: molecular pathophysiology and impact of exercise training. J Cachexia Sarcopenia Muscle. 2015;6(3):197–207. doi: 10.1002/jcsm.12043 26401465 PMC4575550

[54] Ebrahimi SM, Bathaie SZ, Faridi N, Taghikhani M, Nakhjavani M, Faghihzadeh S. L-lysine protects C2C12 myotubes and 3T3-L1 adipocytes against high glucose damages and stresses. PLoS ONE. 2019;14(12):e0225912. doi: 10.1371/journal.pone.0225912 PMC6922410 31856203

[55] Sato T, Ito Y, Nagasawa T. Regulatory effects of the L-lysine metabolites, L-2-aminoadipic acid and L-pipecolic acid, on protein turnover in C2C12 myotubes. Biosci Biotechnol Biochem. 2016;80(11):2168–75. doi: 10.1080/09168451.2016.1210499 27427787

[56] Sato T, Ito Y, Nagasawa T. Dietary L-Lysine Suppresses Autophagic Proteolysis and Stimulates Akt/mTOR Signaling in the Skeletal Muscle of Rats Fed a Low-Protein Diet. J Agric Food Chem. 2015;63(37):8192–8. doi: 10.1021/acs.jafc.5b03811 26366928

[57] Cheng J, Ohsaki Y, Tauchi-Sato K, Fujita A, Fujimoto T. Cholesterol depletion induces autophagy. Biochem Biophys Res Commun. 2006;351(1):246–52. doi: 10.1016/j.bbrc.2006.10.042 17056010

[58] Shapira KE, Shapira G, Schmukler E, Pasmanik-Chor M, Shomron N, Pinkas-Kramarski R, et al. Autophagy is induced and modulated by cholesterol depletion through transcription of autophagy-related genes and attenuation of flux. Cell Death Discov. 2021;7(1):320. doi: 10.1038/s41420-021-00718-3 34716312 PMC8556405

[59] Hanai J, Cao P, Tanksale P, Imamura S, Koshimizu E, Zhao J, et al. The muscle-specific ubiquitin ligase atrogin-1/MAFbx mediates statin-induced muscle toxicity. J Clin Invest. 2007;117(12):3940–51. doi: 10.1172/JCI32741 17992259 PMC2066198

[60] Cao P, Hanai J-I, Tanksale P, Imamura S, Sukhatme VP, Lecker SH. Statin-induced muscle damage and atrogin-1 induction is the result of a geranylgeranylation defect. FASEB J. 2009;23(9):2844–54. doi: 10.1096/fj.08-128843 19406843 PMC2735363

[61] Lagirand-Cantaloube J, Cornille K, Csibi A, Batonnet-Pichon S, Leibovitch MP, Leibovitch SA. Inhibition of atrogin-1/MAFbx mediated MyoD proteolysis prevents skeletal muscle atrophy in vivo. PLoS One. 2009;4(3):e4973. doi: 10.1371/journal.pone.0004973 19319192 PMC2656614

[62] Harford TJ, Kliment G, Shukla GC, Weyman CM. The muscle regulatory transcription factor MyoD participates with p53 to directly increase the expression of the pro-apoptotic Bcl2 family member PUMA. Apoptosis. 2017;22(12):1532–42. doi: 10.1007/s10495-017-1423-x 28918507 PMC5693709

[63] Rahman FA, Quadrilatero J. Mitochondrial Apoptotic Signaling Involvement in Remodeling During Myogenesis and Skeletal Muscle Atrophy. Semin Cell Dev Biol. 2023;143:66–74. doi: 10.1016/j.semcdb.2022.01.011 35241367

[64] Dee K, Freer M, Mei Y, Weyman CM. Apoptosis coincident with the differentiation of skeletal myoblasts is delayed by caspase 3 inhibition and abrogated by MEK-independent constitutive Ras signaling. Cell Death Differ. 2002;9(2):209–18. doi: 10.1038/sj.cdd.4400930 11840171

[65] Bakunts A, Orsi A, Vitale M, Cattaneo A, Lari F, Tadè L, et al. Ratiometric sensing of BiP-client versus BiP levels by the unfolded protein response determines its signaling amplitude. eLife. 2017;6. doi: 10.7554/elife.27518 PMC5792092 29251598

[66] Li J, Lee AS. Stress induction of GRP78/BiP and its role in cancer. Curr Mol Med. 2006;6(1):45–54. doi: 10.2174/156652406775574523 16472112

[67] Garcia D, Shaw RJ. AMPK: Mechanisms of Cellular Energy Sensing and Restoration of Metabolic Balance. Mol Cell. 2017;66(6):789–800. doi: 10.1016/j.molcel.2017.05.032 28622524 PMC5553560

[68] Dellarco VL, Prival MJ. Mutagenicity of nitro compounds in Salmonella typhimurium in the presence of flavin mononucleotide in a preincubation assay. Environ Mol Mutagen. 1989;13(2):116–27. doi: 10.1002/em.2850130206 2645131

[69] Repository ZO. 4-Nitroaniline. MAK Value Documentation, supplement – Translation of the German version from 2020. 2022;7: 0–15.

[70] Alayash AI, Patel RP, Cashon RE. Redox reactions of hemoglobin and myoglobin: biological and toxicological implications. Antioxid Redox Signal. 2001;3(2):313–27. doi: 10.1089/152308601300185250 11396484

[71] Henríquez-Olguín C, Boronat S, Cabello-Verrugio C, Jaimovich E, Hidalgo E, Jensen TE. The Emerging Roles of Nicotinamide Adenine Dinucleotide Phosphate Oxidase 2 in Skeletal Muscle Redox Signaling and Metabolism. Antioxid Redox Signal. 2019;31(18):1371–410. doi: 10.1089/ars.2018.7678 31588777 PMC6859696

[72] Ren Y, Shen H-M. Critical role of AMPK in redox regulation under glucose starvation. Redox Biology. 2019;25:101154. doi: 10.1016/j.redox.2019.101154 30853530 PMC6859544

[73] Liu C-L, Hsu Y-C, Lee J-J, Chen M-J, Lin C-H, Huang S-Y, et al. Targeting the pentose phosphate pathway increases reactive oxygen species and induces apoptosis in thyroid cancer cells. Mol Cell Endocrinol. 2020;499:110595. doi: 10.1016/j.mce.2019.110595 31563469

[74] Lieu EL, Nguyen T, Rhyne S, Kim J. Amino acids in cancer. Exp Mol Med. 2020;52(1):15–30. doi: 10.1038/s12276-020-0375-3 31980738 PMC7000687

[75] Bröer S. Amino acid transporters as targets for cancer therapy: why, where, when, and how. 2020;2. 10.3390/ijms21176156 PMC7503255 32859034

