# Supplementary material for: Lysine p-nitroanilide impairs cellular energetics and potentiates statin-induced cytotoxicity in RD rhabdomyosarcoma cells
Source: PLoS One. 2025 Dec 4;20(12):e0337895. doi: 10.1371/journal.pone.0337895 (PMC12677468; doi:10.1371/journal.pone.0337895)
Supplement: S1 Table — (DOCX) [file pone.0337895.s001.docx]

| S1 Table. List of metabolites included in the untargeted metabolomic analysis | | | |
| --- | --- | --- | --- |
| # | Metabolite | # | Metabolite |
| 1 | Alanine | 12 | Mannitol |
| 2 | Aminoadipic acid | 13 | Myo-inositol |
| 3 | Beta-alanine | 14 | Palmitic acid |
| 4 | Cholesterol | 15 | Pantothenic acid |
| 5 | Citric acid | 16 | Phenylalanine |
| 6 | Fructose1 | 17 | Serine |
| 7 | Fructose2 | 18 | Stearic acid |
| 8 | Galactose | 19 | Talose |
| 9 | Glutamine | 20 | Threonine |
| 10 | Glycerol phosphate | 21 | Tyrosine |
| 11 | Pyruvic/Lactic acid | 22 | Valine |
