# Supplementary material for: Lysine p-nitroanilide impairs cellular energetics and potentiates statin-induced cytotoxicity in RD rhabdomyosarcoma cells
Source: PLoS One. 2025 Dec 4;20(12):e0337895. doi: 10.1371/journal.pone.0337895 (PMC12677468; doi:10.1371/journal.pone.0337895)
Supplement: S1 File — (ZIP) [file pone.0337895.s004.zip › Minimal_data_set/S1_raw_images.pdf]

Raw Blot Images

Figure 1b

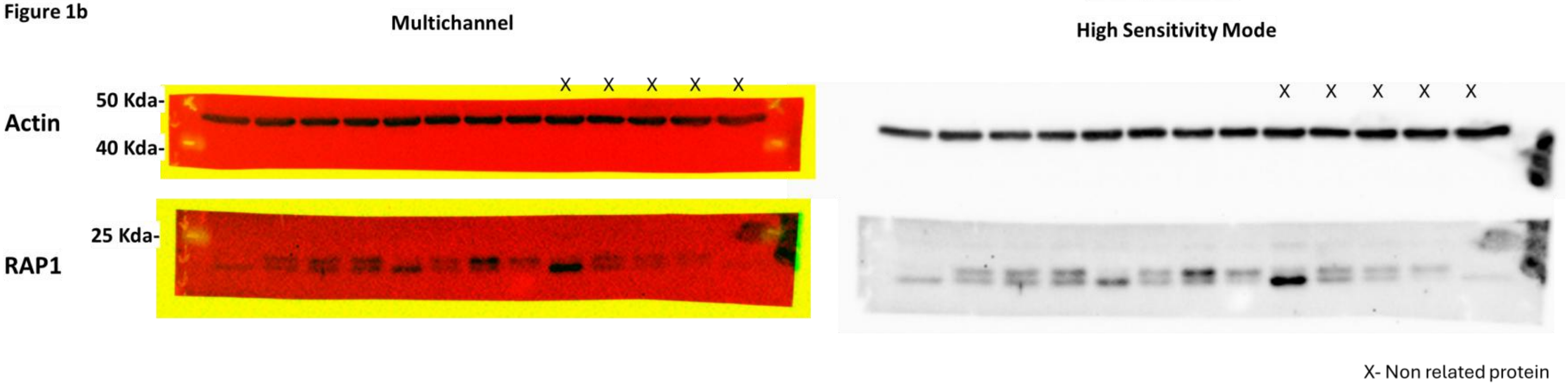

Raw Blot Images Figure 1d

Multichannel | High Sensitivity Mode

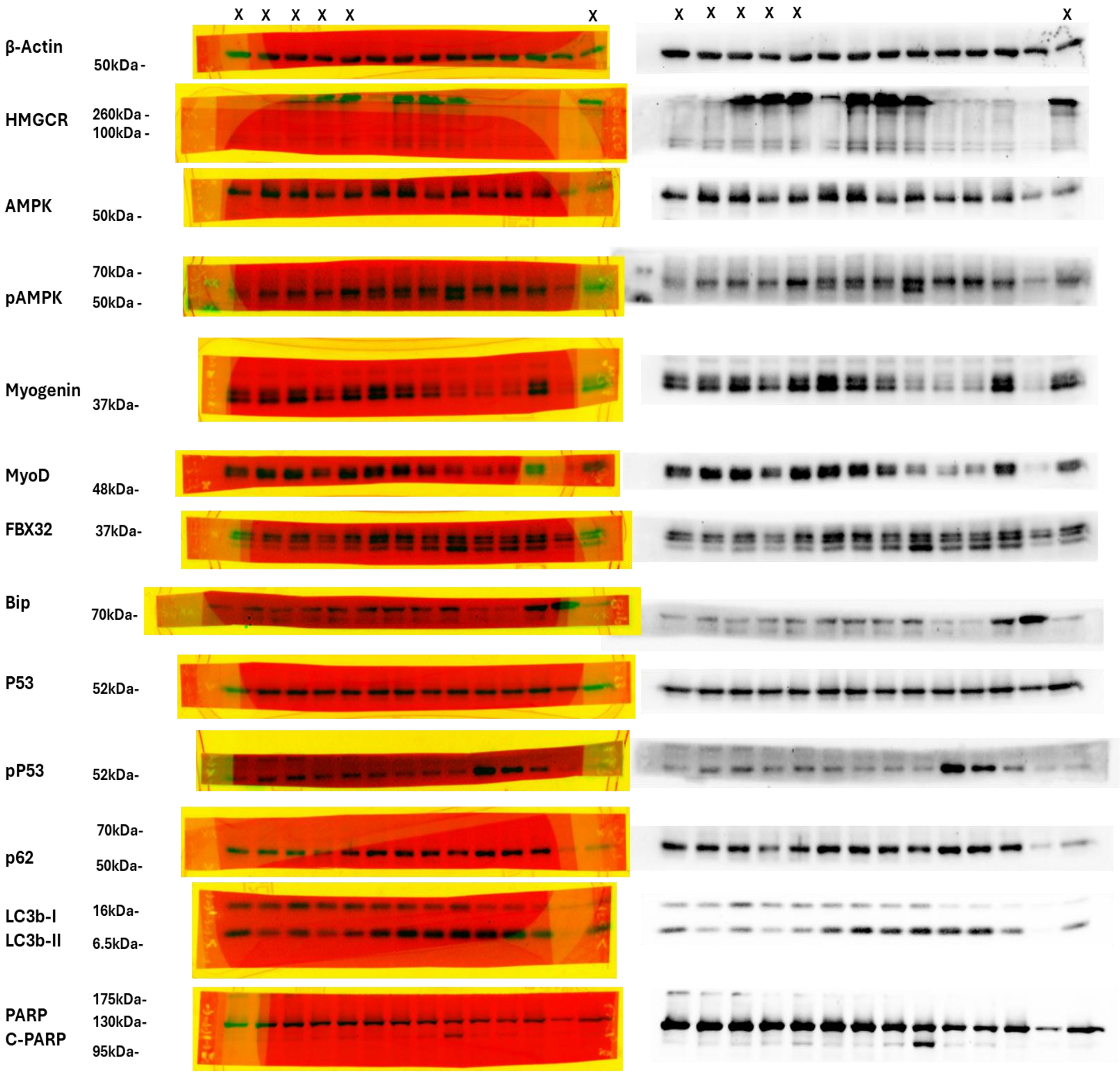

X- Non related protein

Raw Blot Images Figure 2a

Multichannel | High Sensitivity Mode

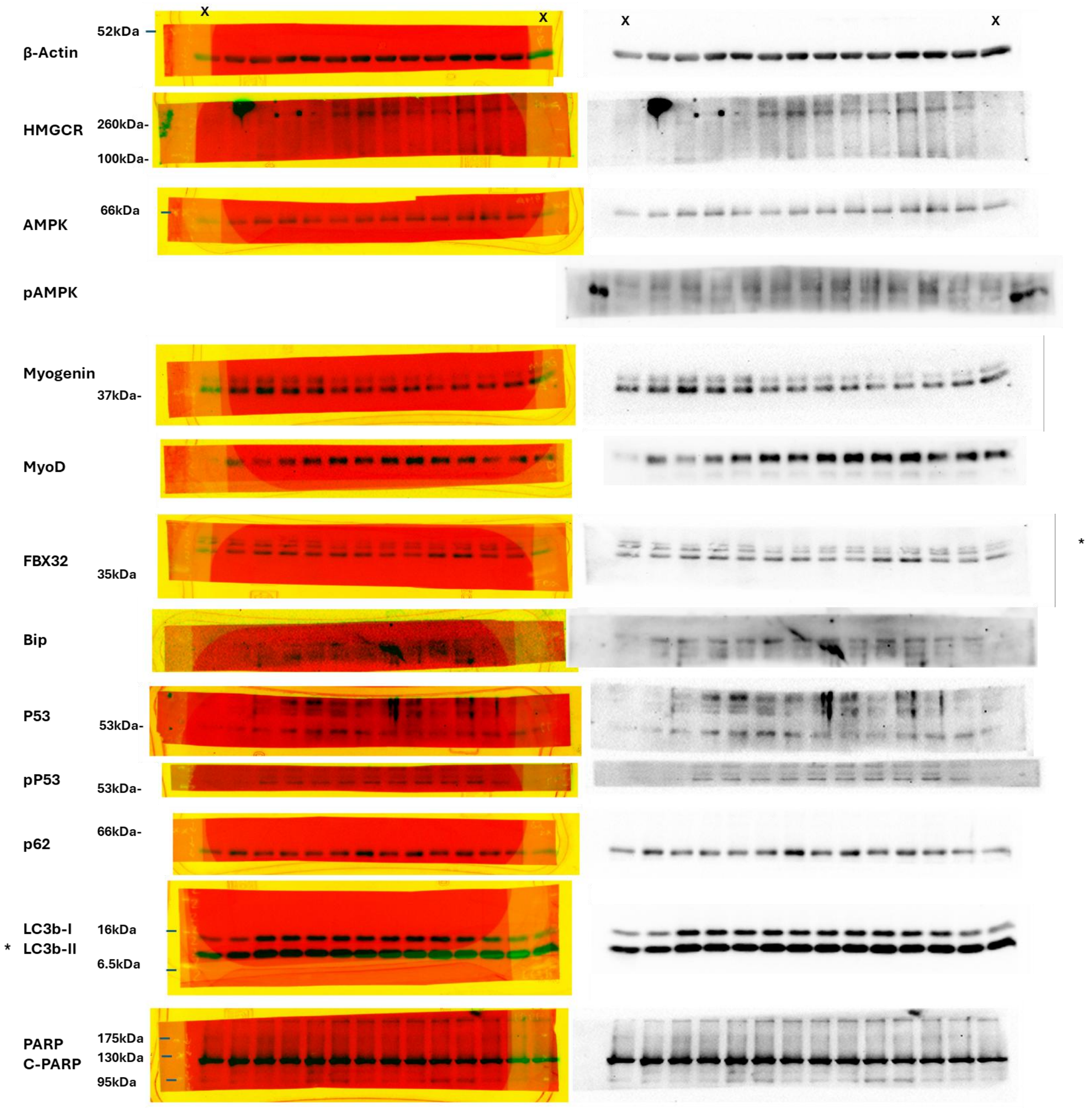

X- Non related protein

Raw Blot Images Figure 2b **Multichannel** **High Sensitivity Mode**

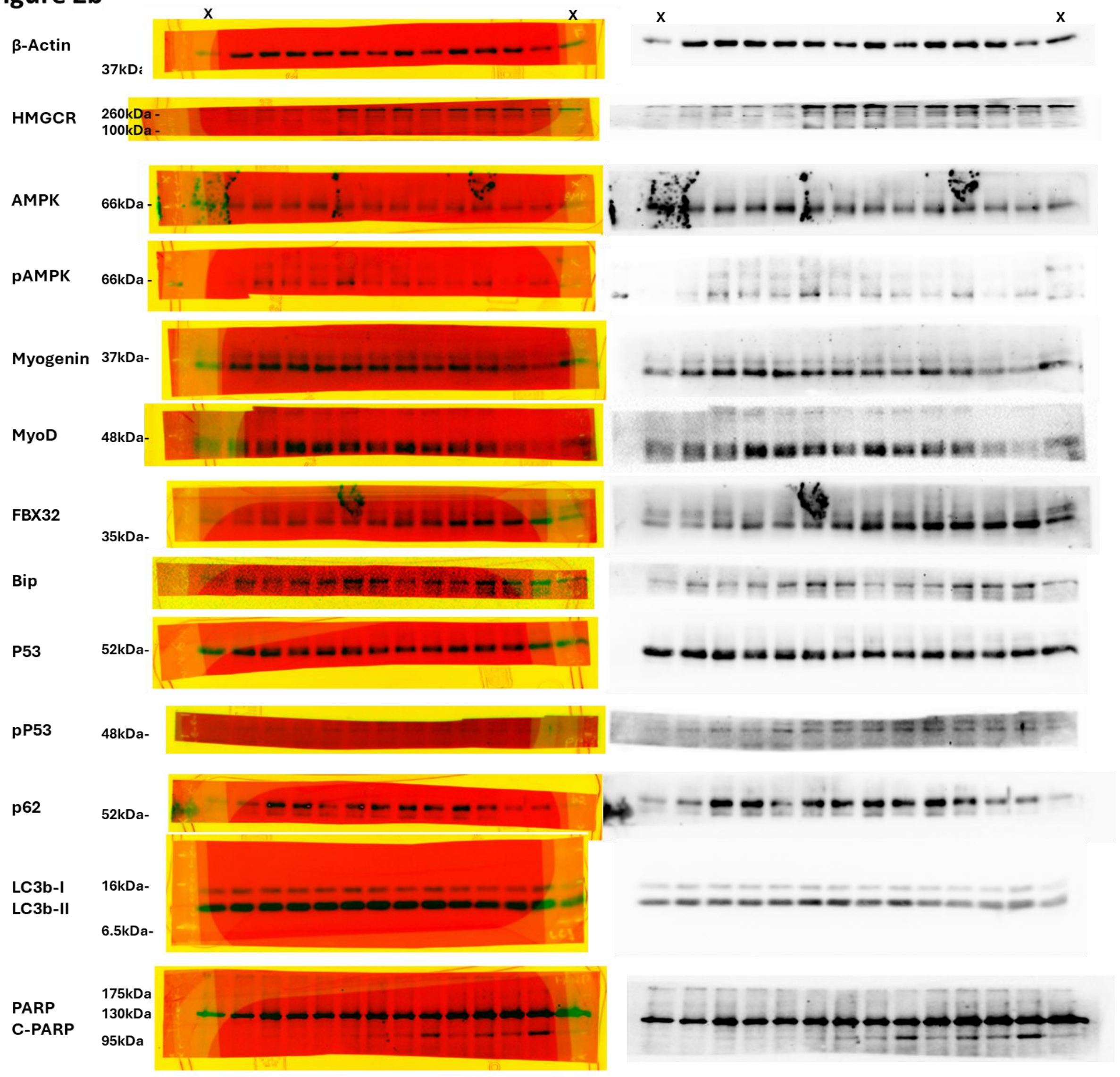

X- Non related protein

Raw Blot Images Figure 2c **Multichannel** **High Sensitivity Mode**

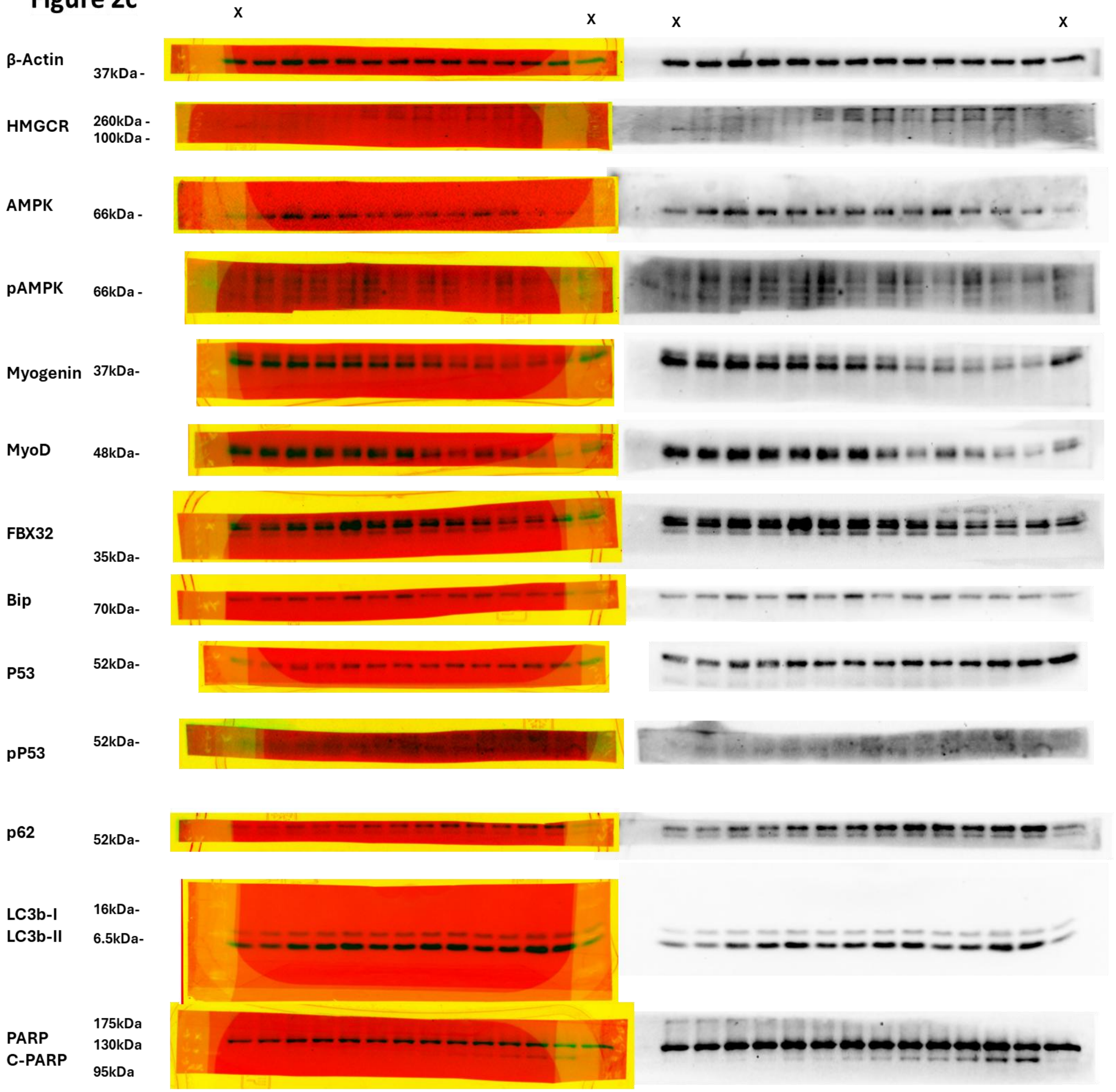

X- Non related protein

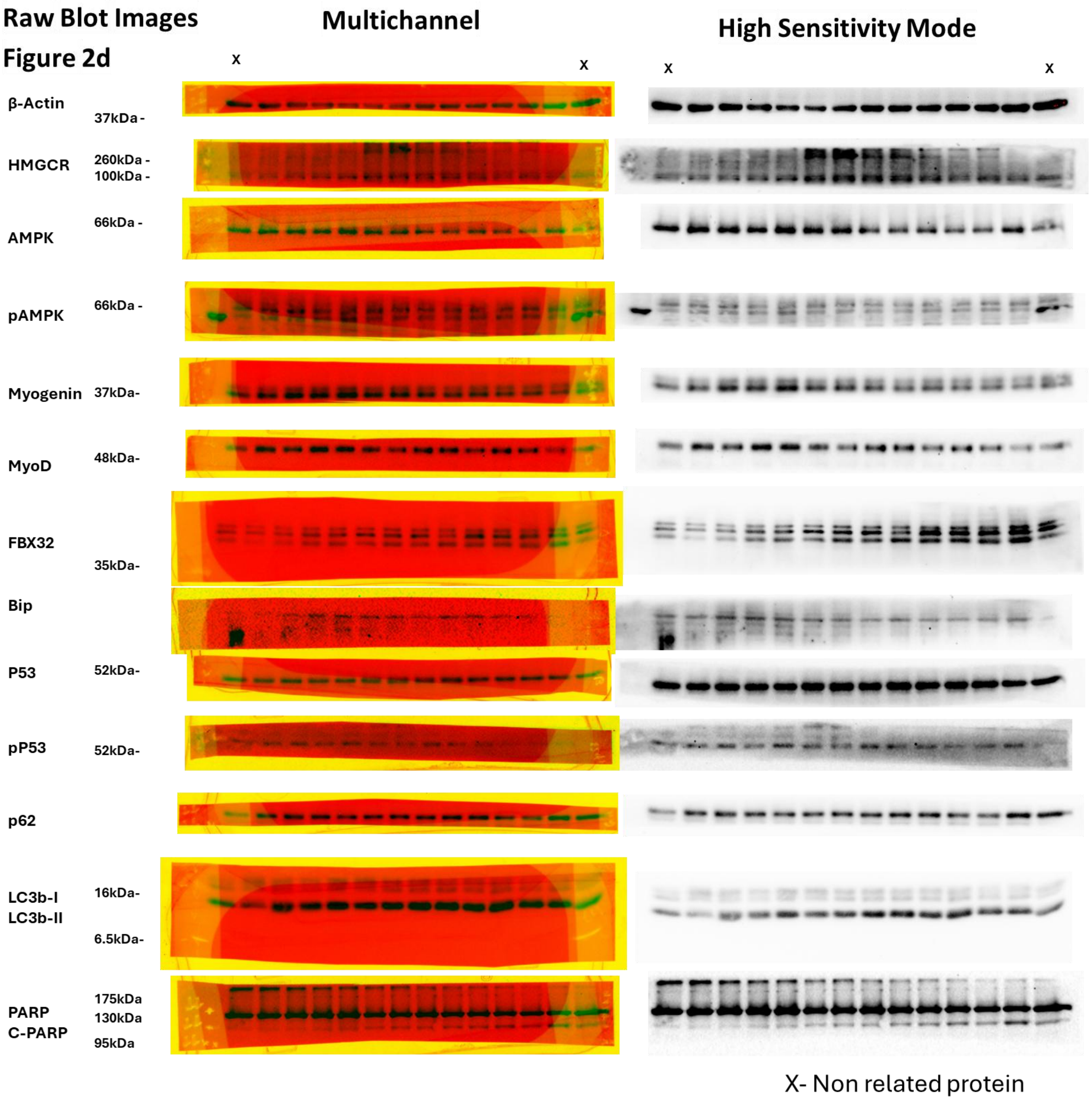
Raw Blot Images
Figure 2d
Multichannel
High Sensitivity Mode
x
x
x
x
β-Actin
37kDa -
HMGCR
260kDa -
100kDa -
AMPK
66kDa -
pAMPK
66kDa -
Myogenin
37kDa-
MyoD
48kDa-
FBX32
35kDa-
Bip
P53
52kDa-
pP53
52kDa-
p62
LC3b-I
LC3b-II
16kDa-
6.5kDa-
PARP
C-PARP
175kDa
130kDa
95kDa
X- Non related protein

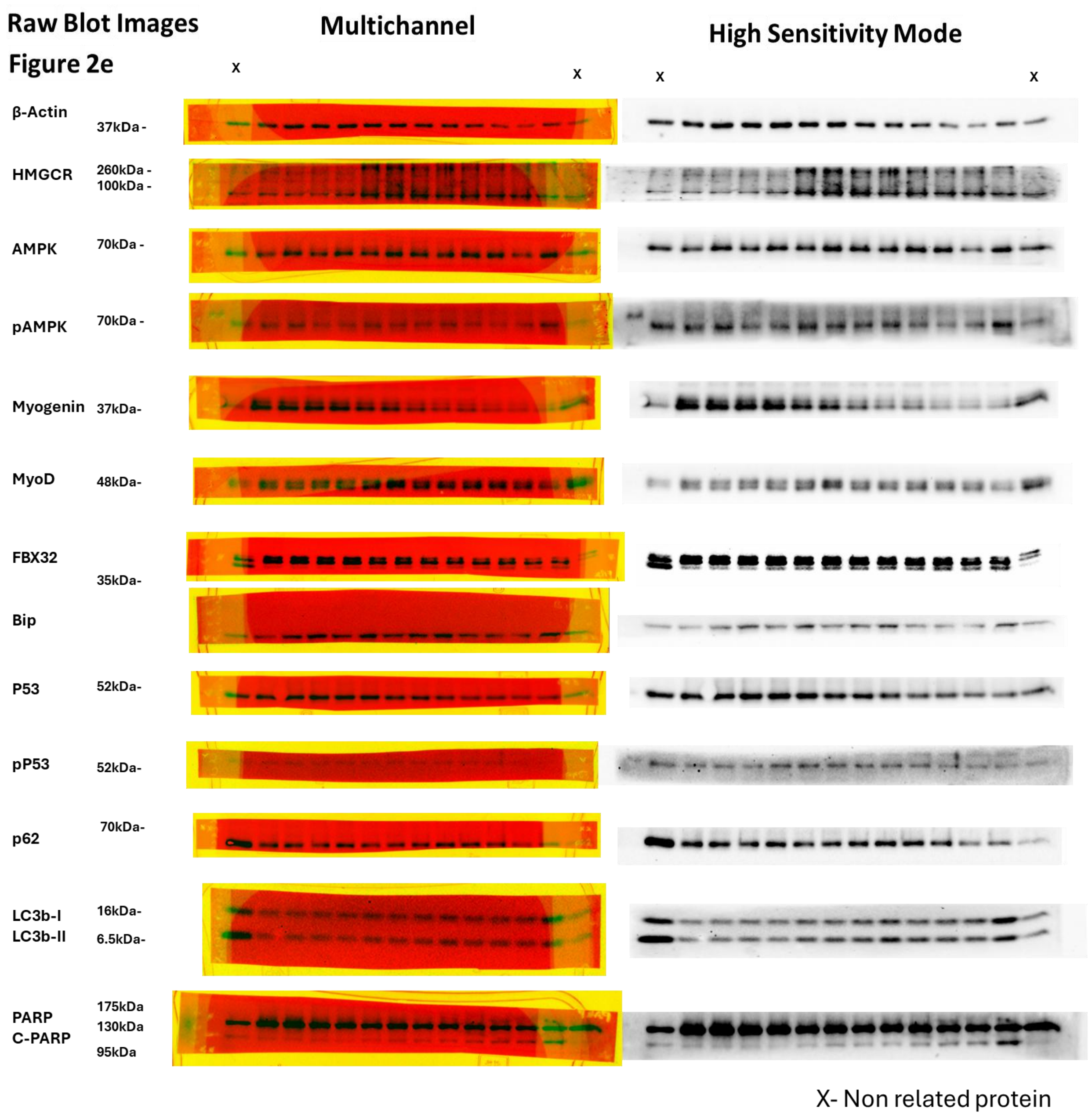
Raw Blot Images
Figure 2e
Multichannel
High Sensitivity Mode
x
x
x
x
β-Actin
37kDa -
HMGCR
260kDa -
100kDa -
AMPK
70kDa -
pAMPK
70kDa -
Myogenin
37kDa-
MyoD
48kDa-
FBX32
35kDa-
Bip
P53
52kDa-
pP53
52kDa-
p62
70kDa-
LC3b-I
LC3b-II
16kDa-
6.5kDa-
PARP
C-PARP
175kDa
130kDa
95kDa
X- Non related protein

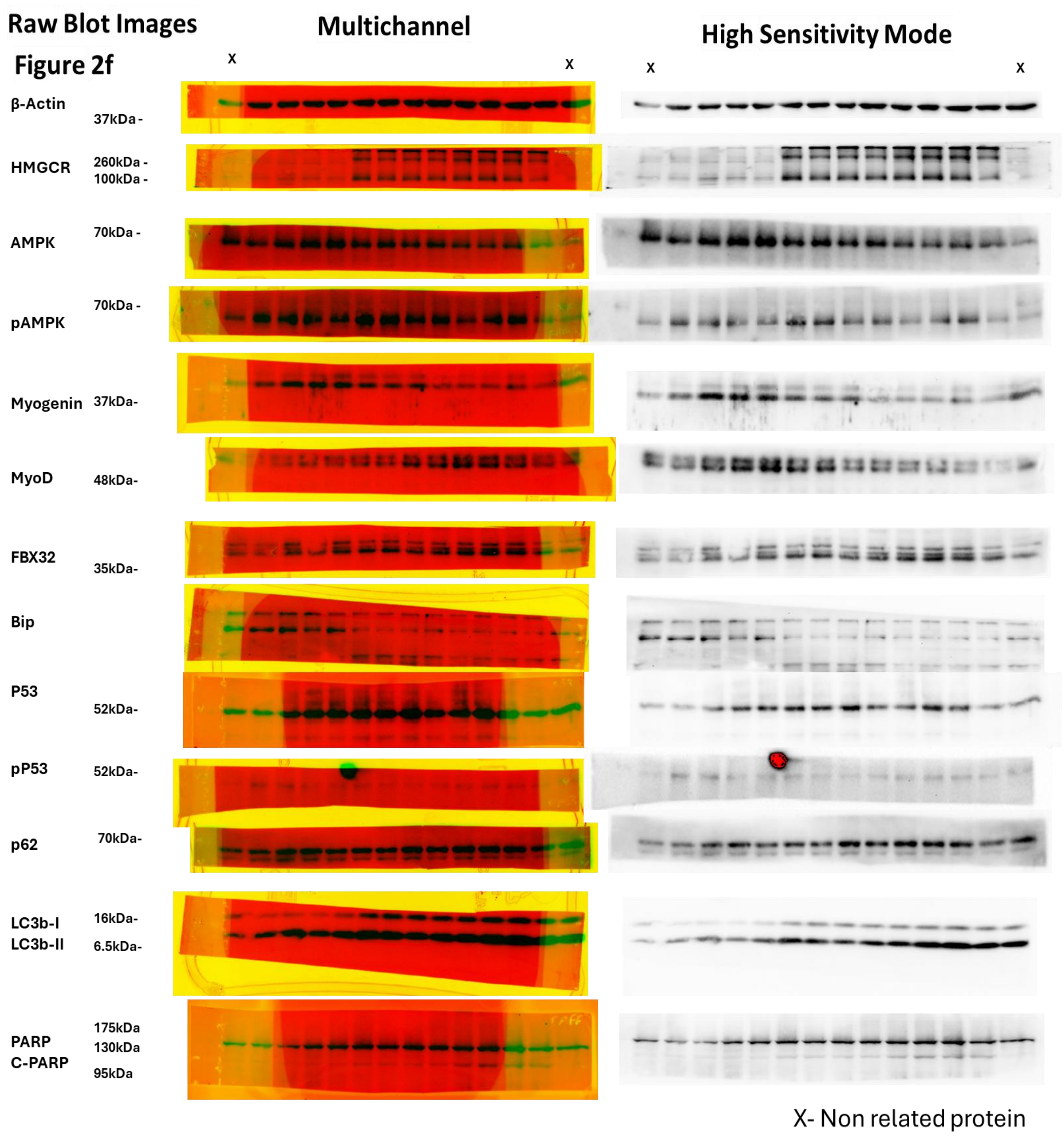
Raw Blot Images
Figure 2f
Multichannel
High Sensitivity Mode
x
x
x
x
β-Actin
37kDa -
HMGCR
260kDa -
100kDa -
AMPK
70kDa -
pAMPK
70kDa -
Myogenin
37kDa-
MyoD
48kDa-
FBX32
35kDa-
Bip
P53
52kDa-
pP53
52kDa-
p62
70kDa-
LC3b-I
LC3b-II
16kDa-
6.5kDa-
PARP
C-PARP
175kDa
130kDa
95kDa
X- Non related protein

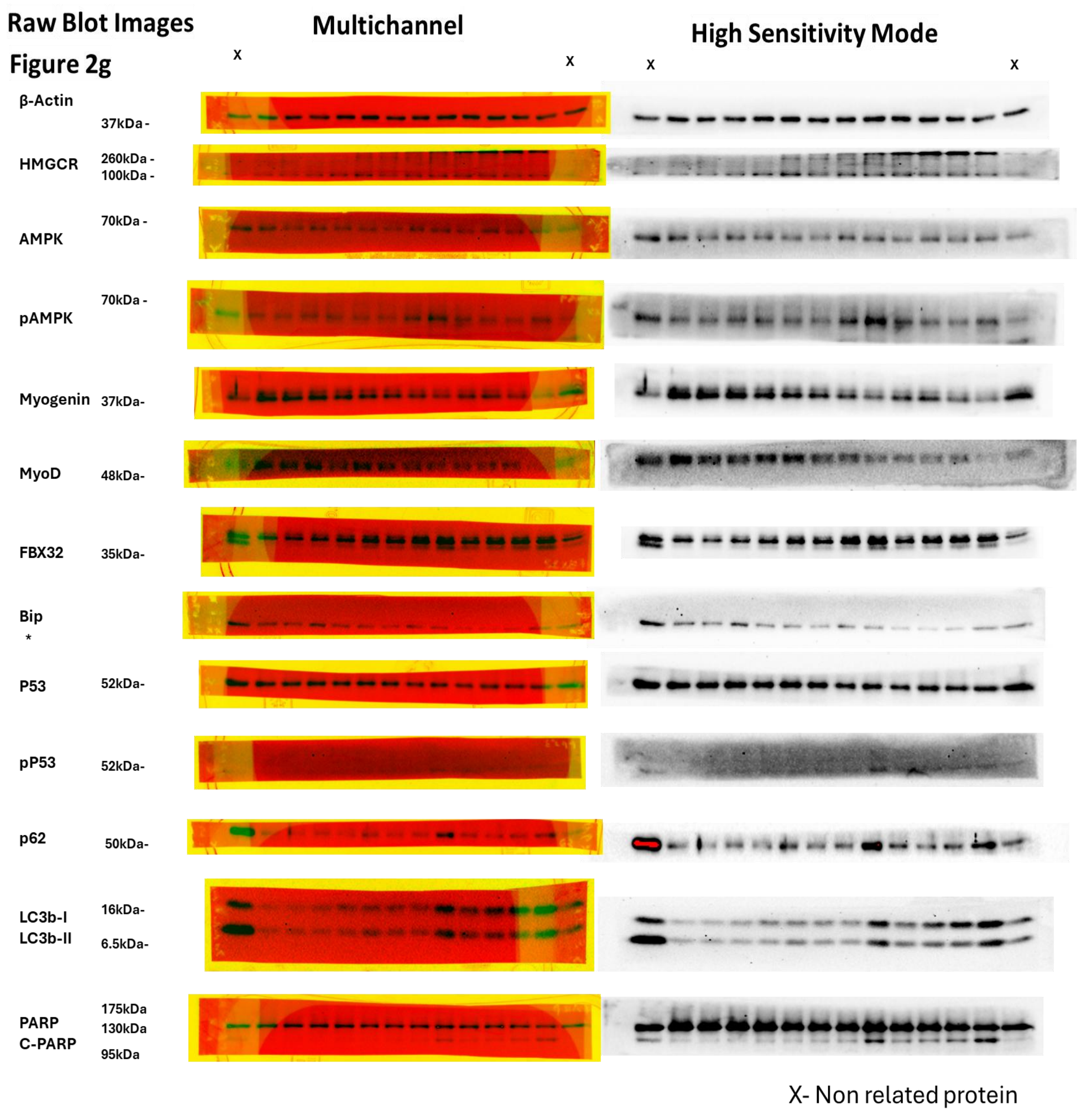
Raw Blot Images
Figure 2g
Multichannel
High Sensitivity Mode
x
x
x
x
β-Actin
37kDa -
HMGCR
260kDa -
100kDa -
AMPK
70kDa -
pAMPK
70kDa -
Myogenin
37kDa-
MyoD
48kDa-
FBX32
35kDa-
Bip
*
P53
52kDa-
pP53
52kDa-
p62
50kDa-
LC3b-I
LC3b-II
16kDa-
6.5kDa-
PARP
C-PARP
175kDa
130kDa
95kDa
X- Non related protein

Raw Blot Images

Figure 2h

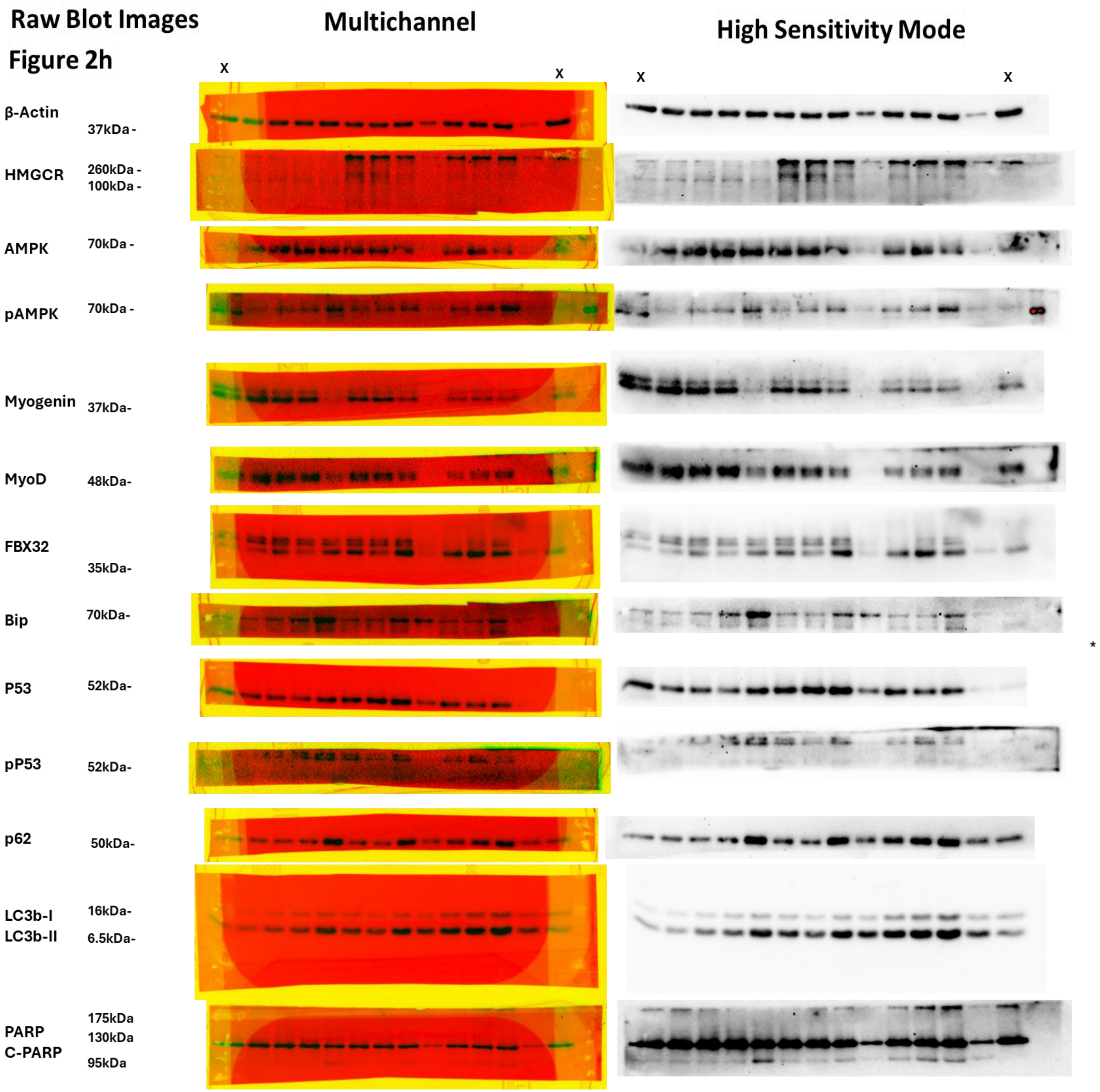

X- Non related protein

Raw Blot Images

Figure 3a

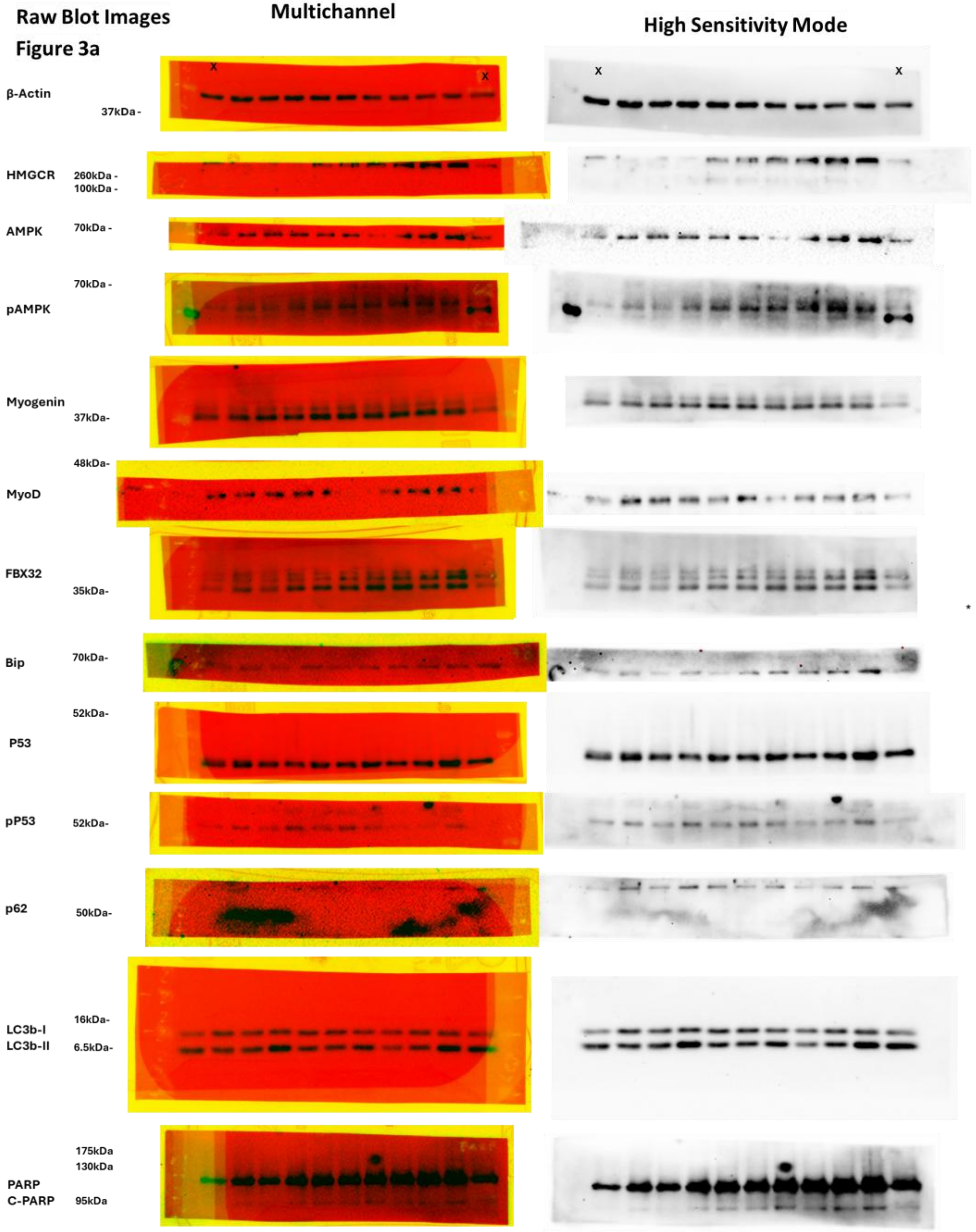

Extraction 1

X- Non related protein

Figure 3c-e

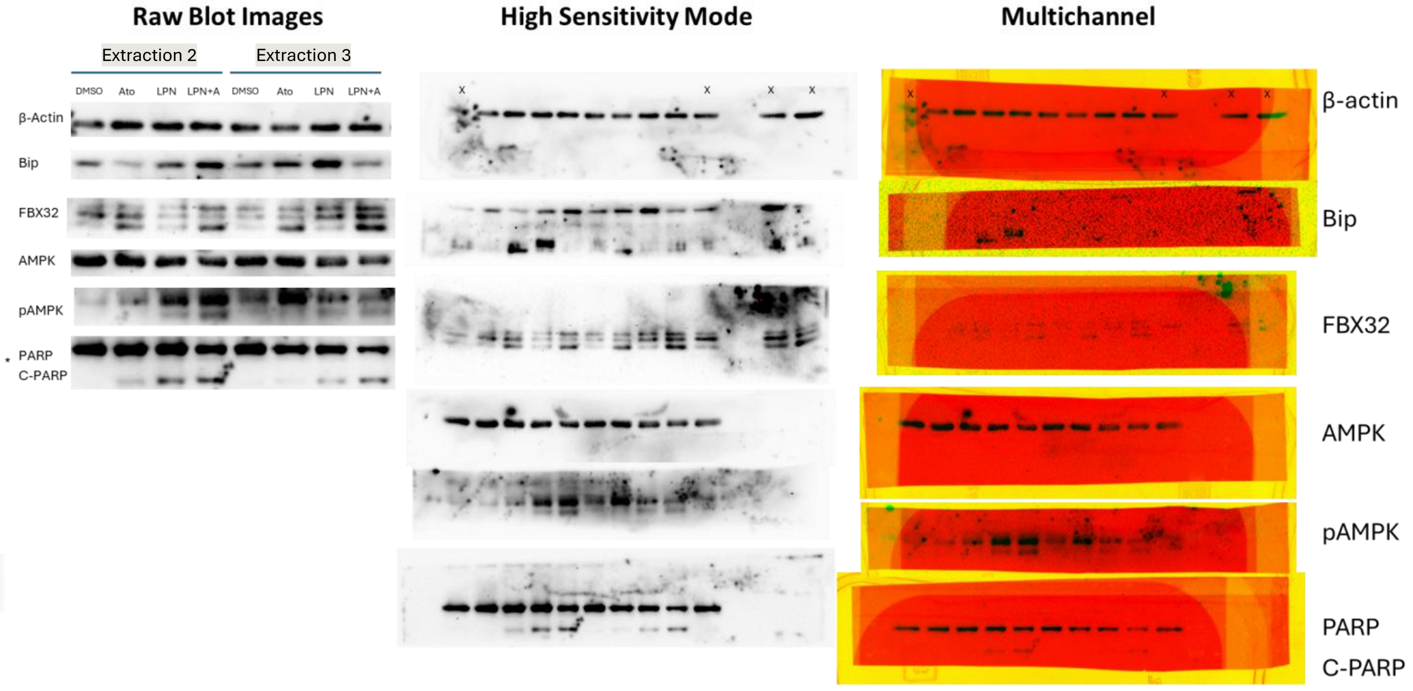

X- Non related protein

Raw Blot Images

Figure 4h

Multichannel

High Sensitivity Mode

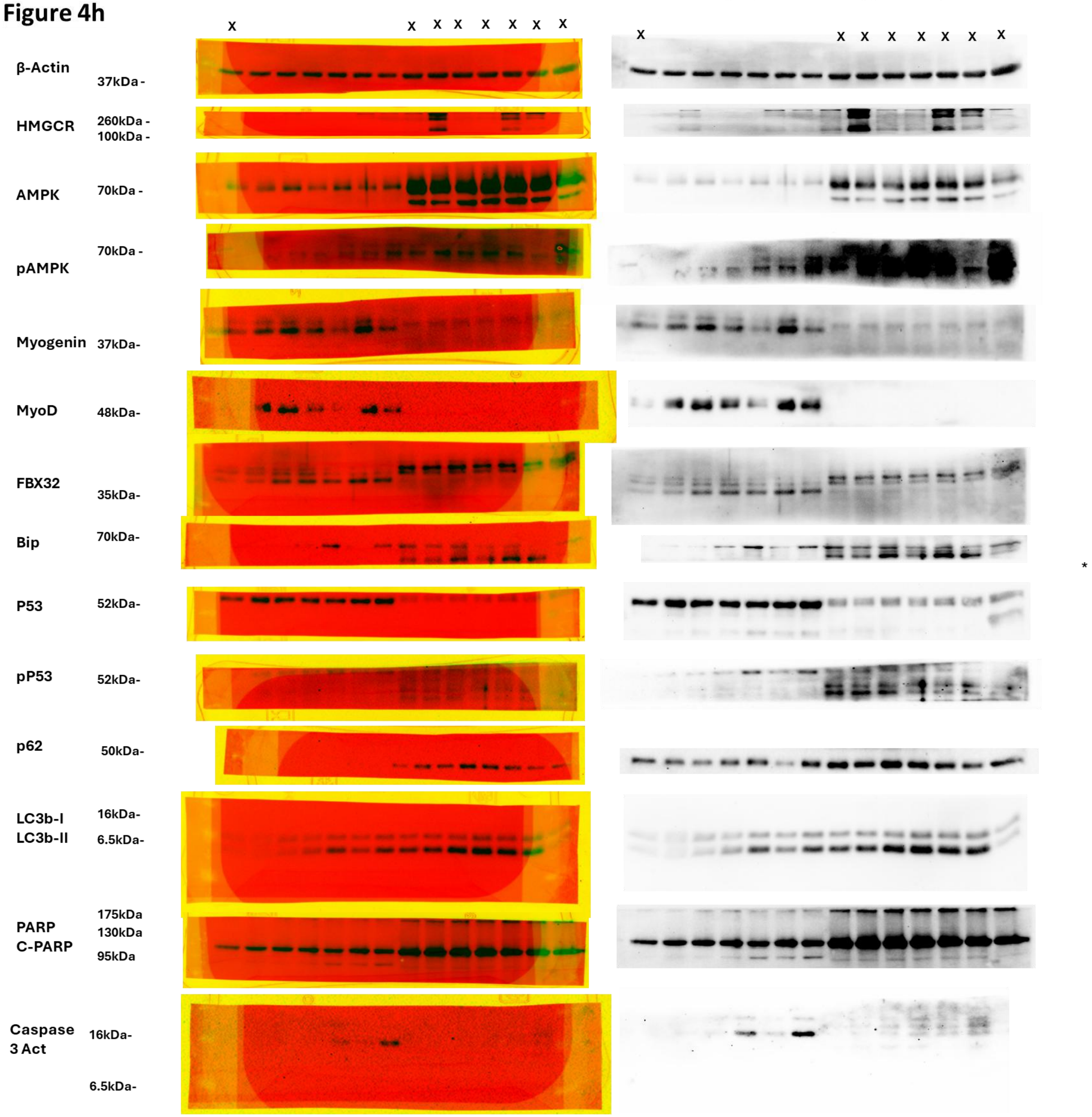

X- Non related protein
